# Supplementary material for: Poor Sensitivity of the MALDI Biotyper® MBT Subtyping Module for Detection of Klebsiella pneumoniae Carbapenemase (KPC) in Klebsiella Species
Source: Antibiotics (Basel). 2023 Sep 20;12(9):1465. doi: 10.3390/antibiotics12091465 (PMC10525285; doi:10.3390/antibiotics12091465)
Supplement: Supplementary file 1 [file antibiotics-12-01465-s001.zip › Supplementary data- Poor sensitivity of the MALDI Biotyper MBT Subtyping Module.pdf]

Table S1. Results of WGS and MALDI-ToF detection in 795 *Klebsiella* isolates.

ISOLATE	KPC PRESENCE by WHOLE GENOME SEQUENCING	KPC TYPE	pKpQIL_*019* STATUS by WHOLE GENOME SEQUENCING	SUBTYPING MODULE IDENTIFICA-TION	SPECIES IDENTIFICATION		REGION *	RESHOOT of DISCREPANT / NOT DETECTED ISOLATE	RESULT of RESHOOT **
					WHOLE GENOME SEQUENCING	MALDI-ToF MASS SPECTROMETRY			
ARLG-7444	No	N/A	NO	Negative	*K. pneumoniae*	*K. pneumoniae*	2	NO	N/A
ARLG-7309	No	N/A	NO	Negative	*K. pneumoniae*	*K. pneumoniae*	3	NO	N/A
ARLG-7314	No	N/A	NO	Negative	*K. pneumoniae*	*K. pneumoniae*	3	NO	N/A
ARLG-7316	No	N/A	NO	Negative	*K. pneumoniae*	*K. pneumoniae*	3	NO	N/A
ARLG-7317	No	N/A	NO	Negative	*K. pneumoniae*	*K. pneumoniae*	3	NO	N/A
ARLG-7318	No	N/A	NO	Negative	*K. pneumoniae*	*K. pneumoniae*	3	NO	N/A
ARLG-7319	No	N/A	NO	Negative	*K. pneumoniae*	*K. pneumoniae*	3	NO	N/A
ARLG-7321	No	N/A	NO	Negative	*K. pneumoniae*	*K. pneumoniae*	3	NO	N/A
ARLG-7322	No	N/A	NO	Negative	*K. pneumoniae*	*K. pneumoniae*	3	NO	N/A
ARLG-7323	No	N/A	NO	Negative	*K. pneumoniae*	*K. pneumoniae*	3	NO	N/A
ARLG-7325	No	N/A	NO	Negative	*K. pneumoniae*	*K. pneumoniae*	3	NO	N/A
ARLG-7326	No	N/A	NO	Negative	*K. pneumoniae*	*K. pneumoniae*	3	NO	N/A
ARLG-7327	No	N/A	NO	Negative	*K. pneumoniae*	*K. pneumoniae*	3	NO	N/A
ARLG-7331	No	N/A	NO	Negative	*K. pneumoniae*	*K. pneumoniae*	3	NO	N/A
ARLG-7332	No	N/A	NO	Negative	*K. pneumoniae*	*K. pneumoniae*	3	NO	N/A
ARLG-7333	No	N/A	NO	Negative	*K. pneumoniae*	*K. pneumoniae*	3	NO	N/A
ARLG-7334	No	N/A	NO	Negative	*K. pneumoniae*	*K. pneumoniae*	3	NO	N/A
ARLG-7335	No	N/A	NO	Negative	*K. pneumoniae*	*K. pneumoniae*	3	NO	N/A
ARLG-7336	No	N/A	NO	Negative	*K. pneumoniae*	*K. pneumoniae*	3	NO	N/A
ARLG-7337	No	N/A	NO	Negative	*K. pneumoniae*	*K. pneumoniae*	3	NO	N/A
ARLG-7338	No	N/A	NO	Negative	*K. pneumoniae*	*K. pneumoniae*	3	NO	N/A
ARLG-7339	No	N/A	NO	Negative	*K. pneumoniae*	*K. pneumoniae*	3	NO	N/A
ARLG-7340	No	N/A	NO	Negative	*K. pneumoniae*	*K. pneumoniae*	3	NO	N/A
ARLG-7341	No	N/A	NO	Negative	*K. pneumoniae*	*K. pneumoniae*	3	NO	N/A

ARLG-7343	No	N/A	NO	Negative	*K. pneumoniae*	*K. pneumoniae*	3	NO	N/A
ARLG-7344	No	N/A	NO	Negative	*K. pneumoniae*	*K. pneumoniae*	3	NO	N/A
ARLG-7345	No	N/A	NO	Negative	*K. pneumoniae*	*K. pneumoniae*	3	NO	N/A
ARLG-7346	No	N/A	NO	Negative	*K. pneumoniae*	*K. pneumoniae*	3	NO	N/A
ARLG-7347	No	N/A	NO	Negative	*K. pneumoniae*	*K. pneumoniae*	3	NO	N/A
ARLG-7245	No	N/A	NO	Negative	*K. pneumoniae*	*K. pneumoniae*	2	NO	N/A
ARLG-7246	No	N/A	NO	Negative	*K. pneumoniae*	*K. pneumoniae*	2	NO	N/A
ARLG-7504	No	N/A	NO	Negative	*K. pneumoniae*	*K. pneumoniae*	2	NO	N/A
ARLG-7348	No	N/A	NO	Negative	*K. pneumoniae*	*K. pneumoniae*	3	NO	N/A
ARLG-7349	No	N/A	NO	Negative	*K. pneumoniae*	*K. pneumoniae*	3	NO	N/A
ARLG-7350	No	N/A	NO	Negative	*K. pneumoniae*	*K. pneumoniae*	3	NO	N/A
ARLG-7353	No	N/A	NO	Negative	*K. pneumoniae*	*K. pneumoniae*	3	NO	N/A
ARLG-7356	No	N/A	NO	Negative	*K. pneumoniae*	*K. pneumoniae*	3	NO	N/A
ARLG-7359	No	N/A	NO	Negative	*K. pneumoniae*	*K. pneumoniae*	3	NO	N/A
ARLG-7360	No	N/A	NO	Negative	*K. pneumoniae*	*K. pneumoniae*	3	NO	N/A
ARLG-7342	No	N/A	NO	Negative	*K. pneumoniae*	*K. pneumoniae*	3	NO	N/A
ARLG-7354	No	N/A	NO	Negative	*K. pneumoniae*	*K. pneumoniae*	3	NO	N/A
ARLG-7228	Yes	KPC2	NO	Negative	*K. pneumoniae*	*K. pneumoniae*	2	NO	N/A
ARLG-7440	No	N/A	NO	Negative	*K. pneumoniae*	*K. pneumoniae*	2	NO	N/A
ARLG-7683	No	N/A	NO	Negative	*K. pneumoniae*	*K. pneumoniae*	1	NO	N/A
ARLG-7612	No	N/A	NO	Negative	*K. pneumoniae*	*K. pneumoniae*	1	NO	N/A
ARLG-8110	No	N/A	NO	Negative	*K. pneumoniae*	*K. pneumoniae*	1	NO	N/A
ARLG-7687	No	N/A	NO	Negative	*K. pneumoniae*	*K. pneumoniae*	1	NO	N/A
ARLG-7568	No	N/A	NO	Negative	*K. pneumoniae*	*K. pneumoniae*	1	NO	N/A
ARLG-7794	Yes	KPC2	NO	Negative	*K. pneumoniae*	*K. pneumoniae*	1	NO	N/A
ARLG-7533	No	N/A	NO	Negative	*K. pneumoniae*	*K. pneumoniae*	1	NO	N/A
ARLG-8120	No	N/A	NO	Negative	*K. pneumoniae*	*K. pneumoniae*	1	NO	N/A
ARLG-8122	No	N/A	NO	Negative	*K. pneumoniae*	*K. pneumoniae*	1	NO	N/A
ARLG-8034	No	N/A	NO	Negative	*K. pneumoniae*	*K. pneumoniae*	1	NO	N/A
ARLG-8128	No	N/A	NO	Negative	*K. variicola*	*K. variicola*	1	NO	N/A

ARLG-7913	No	N/A	NO	Negative	*K. pneumoniae*	*K. pneumoniae*	1	NO	N/A
ARLG-7815	No	N/A	NO	Negative	*K. pneumoniae*	*K. pneumoniae*	1	NO	N/A
ARLG-7703	No	N/A	NO	Negative	*K. pneumoniae*	*K. pneumoniae*	1	NO	N/A
ARLG-7546	No	N/A	NO	Negative	*K. pneumoniae*	*K. pneumoniae*	1	NO	N/A
ARLG-7667	No	N/A	NO	Negative	*K. pneumoniae*	*K. pneumoniae*	1	NO	N/A
ARLG-7724	Yes	KPC2	NO	Negative	*K. pneumoniae*	*K. pneumoniae*	1	NO	N/A
ARLG-7823	No	N/A	NO	Negative	*K. variicola*	*K. variicola*	1	NO	N/A
ARLG-8134	Yes	KPC2	NO	Negative	*K. pneumoniae*	*K. pneumoniae*	1	NO	N/A
ARLG-8082	No	N/A	NO	Negative	*K. pneumoniae*	*K. pneumoniae*	1	NO	N/A
ARLG-7507	Yes	KPC3	NO	Negative	*K. pneumoniae*	*K. pneumoniae*	1	NO	N/A
ARLG-8045	No	N/A	NO	Negative	*K. pneumoniae*	*K. pneumoniae*	1	NO	N/A
ARLG-8064	No	N/A	NO	Negative	*K. pneumoniae*	*K. pneumoniae*	1	YES	3
ARLG-8068	No	N/A	NO	Negative	*K. pneumoniae*	*K. pneumoniae*	1	NO	N/A
ARLG-7668	No	N/A	NO	Negative	*K. pneumoniae*	*K. pneumoniae*	1	NO	N/A
ARLG-7671	Yes	KPC2<br>8	NO	Negative	*K. pneumoniae*	*K. pneumoniae*	1	NO	N/A
ARLG-7807	Yes	KPC3<br>4	NO	Negative	*K. pneumoniae*	*K. pneumoniae*	1	NO	N/A
ARLG-7810	No	N/A	NO	Negative	*K. pneumoniae*	*K. pneumoniae*	1	NO	N/A
ARLG-7818	No	N/A	NO	Negative	*K. pneumoniae*	*K. pneumoniae*	1	NO	N/A
ARLG-7825	No	N/A	NO	Negative	*K. pneumoniae*	*K. pneumoniae*	1	NO	N/A
ARLG-7888	No	N/A	NO	Negative	*K. pneumoniae*	*K. pneumoniae*	1	NO	N/A
ARLG-8022	No	N/A	NO	Negative	*K. pneumoniae*	*K. pneumoniae*	1	NO	N/A
ARLG-8039	No	N/A	NO	Negative	*K. pneumoniae*	*K. pneumoniae*	1	NO	N/A
ARLG-7905	No	N/A	NO	Negative	*K. pneumoniae*	*K. pneumoniae*	1	NO	N/A
ARLG-7578	No	N/A	NO	Negative	*K. pneumoniae*	*K. pneumoniae*	1	NO	N/A
ARLG-7615	No	N/A	NO	Negative	*K. pneumoniae*	*K. pneumoniae*	1	NO	N/A
ARLG-7640	No	N/A	NO	Negative	*K. pneumoniae*	*K. pneumoniae*	1	NO	N/A
ARLG-7645	No	N/A	NO	Negative	*K. pneumoniae*	*K. pneumoniae*	1	NO	N/A
ARLG-7929	No	N/A	NO	Negative	*K. pneumoniae*	*K. pneumoniae*	1	NO	N/A

ARLG-7938	No	N/A	NO	Negative	*K. pneumoniae*	*K. pneumoniae*	1	NO	N/A
ARLG-7835	No	N/A	NO	Negative	*K. pneumoniae*	*K. pneumoniae*	1	NO	N/A
ARLG-6445	No	N/A	NO	Negative	*K. pneumoniae*	*K. pneumoniae*	4	NO	N/A
ARLG-6450	No	N/A	NO	Negative	*K. pneumoniae*	*K. pneumoniae*	4	NO	N/A
ARLG-6452	No	N/A	NO	Negative	*K. pneumoniae*	*K. pneumoniae*	4	NO	N/A
ARLG-6511	No	N/A	NO	Negative	*K. pneumoniae*	*K. pneumoniae*	4	NO	N/A
ARLG-6514	No	N/A	NO	Negative	*K. pneumoniae*	*K. pneumoniae*	4	NO	N/A
ARLG-6518	No	N/A	NO	Negative	*K. pneumoniae*	*K. pneumoniae*	4	NO	N/A
ARLG-6520	No	N/A	NO	Negative	*K. pneumoniae*	*K. pneumoniae*	4	NO	N/A
ARLG-6473	No	N/A	NO	Negative	*K. pneumoniae*	*K. pneumoniae*	4	NO	N/A
ARLG-6480	No	N/A	NO	Negative	*K. pneumoniae*	*K. pneumoniae*	4	NO	N/A
ARLG-6490	No	N/A	NO	Negative	*K. pneumoniae*	*K. pneumoniae*	4	NO	N/A
ARLG-6493	No	N/A	NO	Negative	*K. pneumoniae*	*K. pneumoniae*	4	NO	N/A
ARLG-6537	No	N/A	NO	Negative	*K. pneumoniae*	*K. pneumoniae*	4	NO	N/A
ARLG-6547	No	N/A	NO	Negative	*K. pneumoniae*	*K. pneumoniae*	4	NO	N/A
ARLG-8087-P	Yes	KPC2	NO	Negative	*K. pneumoniae*	*K. pneumoniae*	1	NO	N/A
ARLG-8091-P	Yes	KPC3	NO	Negative	*K. pneumoniae*	*K. pneumoniae*	1	NO	N/A
ARLG-8099-P	Yes	KPC3	YES	Negative	*K. pneumoniae*	*K. pneumoniae*	1	NO	N/A
ARLG-8101-P	Yes	KPC3	NO	Negative	*K. pneumoniae*	*K. pneumoniae*	1	NO	N/A
ARLG-8095-P	No	N/A	NO	Negative	*K. pneumoniae*	*K. pneumoniae*	1	YES	1
ARLG-8107-P	Yes	KPC3	YES	Positive	*K. pneumoniae*	*K. pneumoniae*	1	NO	N/A
ARLG-7711-P	Yes	KPC3	NO	Negative	*K. pneumoniae*	*K. pneumoniae*	1	NO	N/A
ARLG-7713-P	Yes	KPC3	NO	Negative	*K. pneumoniae*	*K. pneumoniae*	1	NO	N/A
ARLG-7712-P	Yes	KPC3	NO	Negative	*K. pneumoniae*	*K. pneumoniae*	1	NO	N/A

ARLG-7509-P	Yes	KPC3	NO	Negative	*K. pneumoniae*	*K. pneumoniae*	1	NO	N/A
ARLG-7510-P	Yes	KPC2	NO	Negative	*K. pneumoniae*	*K. pneumoniae*	1	NO	N/A
ARLG-7513-P	Yes	KPC3	YES	Positive	*K. pneumoniae*	*K. pneumoniae*	1	NO	N/A
ARLG-7517-P	Yes	KPC2	NO	Negative	*K. pneumoniae*	*K. pneumoniae*	1	NO	N/A
ARLG-7520-P	Yes	KPC2	YES	Positive	*K. pneumoniae*	*K. pneumoniae*	1	NO	N/A
ARLG-7521-P	Yes	KPC3	NO	Negative	*K. pneumoniae*	*K. pneumoniae*	1	NO	N/A
ARLG-7522-P	Yes	KPC2	NO	Negative	*K. pneumoniae*	*K. pneumoniae*	1	NO	N/A
ARLG-7525-P	Yes	KPC2	YES	Positive	*K. pneumoniae*	*K. pneumoniae*	1	NO	N/A
ARLG-7527-P	Yes	KPC2	NO	Negative	*K. pneumoniae*	*K. pneumoniae*	1	NO	N/A
ARLG-7528-P	Yes	KPC2	NO	Negative	*K. pneumoniae*	*K. pneumoniae*	1	NO	N/A
ARLG-7532-P	Yes	KPC3	NO	Negative	*K. pneumoniae*	*K. pneumoniae*	1	NO	N/A
ARLG-7829-P	Yes	KPC2	YES	Positive	*K. pneumoniae*	*K. pneumoniae*	1	NO	N/A
ARLG-7832-P	Yes	KPC3	NO	Negative	*K. pneumoniae*	*K. pneumoniae*	1	NO	N/A
ARLG-7839-P	Yes	KPC2	YES	Positive	*K. pneumoniae*	*K. pneumoniae*	1	NO	N/A
ARLG-8109-P	Yes	KPC3	NO	Negative	*K. pneumoniae*	*K. pneumoniae*	1	NO	N/A
ARLG-8111-P	Yes	KPC3	YES	Negative	*K. pneumoniae*	*K. pneumoniae*	1	NO	N/A
ARLG-8117-P	No	N/A	NO	Negative	*K. pneumoniae*	*K. pneumoniae*	1	NO	N/A
ARLG-8127-P	Yes	KPC3	NO	Negative	*K. pneumoniae*	*K. pneumoniae*	1	NO	N/A

ARLG-7714-P	Yes	KPC3	NO	Negative	*K. pneumoniae*	*K. pneumoniae*	1	NO	N/A
ARLG-7716-P	Yes	KPC3	NO	Negative	*K. pneumoniae*	*K. pneumoniae*	1	NO	N/A
ARLG-7717-P	Yes	KPC3	NO	Negative	*K. pneumoniae*	*K. pneumoniae*	1	NO	N/A
ARLG-7718-P	Yes	KPC3	NO	Negative	*K. pneumoniae*	*K. pneumoniae*	1	NO	N/A
ARLG-7535-P	Yes	KPC2	NO	Negative	*K. pneumoniae*	*K. pneumoniae*	1	NO	N/A
ARLG-7536-P	Yes	KPC2	YES	Positive	*K. pneumoniae*	*K. pneumoniae*	1	NO	N/A
ARLG-7537-P	Yes	KPC3	YES	Positive	*K. pneumoniae*	*K. pneumoniae*	1	NO	N/A
ARLG-7542-P	Yes	KPC3	NO	Negative	*K. variicola*	*K. variicola*	1	NO	N/A
ARLG-7544-P	Yes	KPC2	YES	Positive	*K. pneumoniae*	*K. pneumoniae*	1	NO	N/A
ARLG-7547-P	Yes	KPC3	YES	Positive	*K. pneumoniae*	*K. pneumoniae*	1	NO	N/A
ARLG-7548-P	Yes	KPC2	NO	Negative	*K. pneumoniae*	*K. pneumoniae*	1	NO	N/A
ARLG-7550-P	Yes	KPC2	YES	Positive	*K. pneumoniae*	*K. pneumoniae*	1	NO	N/A
ARLG-7551-P	Yes	KPC2	YES	Negative	*K. pneumoniae*	*K. pneumoniae*	1	NO	N/A
ARLG-7552-P	Yes	KPC2	NO	Negative	*K. pneumoniae*	*K. pneumoniae*	1	NO	N/A
ARLG-7553-P	Yes	KPC3	YES	Positive	*K. pneumoniae*	*K. pneumoniae*	1	NO	N/A
ARLG-7529-P	Yes	KPC2	YES	Positive	*K. pneumoniae*	*K. pneumoniae*	1	NO	N/A
ARLG-7847-P	Yes	KPC3	NO	Negative	*K. pneumoniae*	*K. pneumoniae*	1	NO	N/A
ARLG-7849-P	Yes	KPC3	NO	Negative	*K. pneumoniae*	*K. pneumoniae*	1	NO	N/A

ARLG-7799-P	Yes	KPC3	YES	Negative	*K. pneumoniae*	*K. pneumoniae*	1	YES	3
ARLG-7801-P	Yes	KPC2	NO	Negative	*K. pneumoniae*	*K. pneumoniae*	1	NO	N/A
ARLG-8135-P	Yes	KPC3	NO	Negative	*K. pneumoniae*	*K. pneumoniae*	1	NO	N/A
ARLG-7541-P	Yes	KPC2	NO	Negative	*K. pneumoniae*	*K. pneumoniae*	1	NO	N/A
ARLG-7837-P	Yes	KPC2	YES	Positive	*K. pneumoniae*	*K. pneumoniae*	1	NO	N/A
ARLG-7851-P	Yes	KPC3	NO	Negative	*K. pneumoniae*	*K. pneumoniae*	1	YES	1
ARLG-7675-P	Yes	KPC3	NO	Negative	*K. pneumoniae*	*K. pneumoniae*	1	NO	N/A
ARLG-7576-P	Yes	KPC2	YES	Positive	*K. pneumoniae*	*K. pneumoniae*	1	NO	N/A
ARLG-7556-P	Yes	KPC2	YES	Positive	*K. pneumoniae*	*K. pneumoniae*	1	YES	2
ARLG-7582-P	Yes	KPC2	YES	Positive	*K. pneumoniae*	*K. pneumoniae*	1	NO	N/A
ARLG-7957-P	Yes	KPC3	NO	Negative	*K. pneumoniae*	*K. pneumoniae*	1	NO	N/A
ARLG-7584-P	No	N/A	NO	Negative	*K. pneumoniae*	*K. pneumoniae*	1	NO	N/A
ARLG-7988-P	Yes	KPC3	NO	Negative	*K. pneumoniae*	*K. pneumoniae*	1	NO	N/A
ARLG-7591-P	Yes	KPC2	NO	Negative	*K. pneumoniae*	*K. pneumoniae*	1	NO	N/A
ARLG-7924-P	Yes	KPC3	NO	Negative	*K. pneumoniae*	*K. pneumoniae*	1	NO	N/A
ARLG-7557-P	Yes	KPC3	NO	Negative	*K. pneumoniae*	*K. pneumoniae*	1	NO	N/A
ARLG-7596-P	Yes	KPC2	YES	Positive	*K. pneumoniae*	*K. pneumoniae*	1	YES	2
ARLG-7560-P	Yes	KPC2	YES	Positive	*K. pneumoniae*	*K. pneumoniae*	1	NO	N/A

ARLG-7889-P	Yes	KPC3	NO	Negative	*K. pneumoniae*	*K. pneumoniae*	1	NO	N/A
ARLG-8025-P	Yes	KPC3	NO	Negative	*K. pneumoniae*	*K. pneumoniae*	1	NO	N/A
ARLG-7565-P	Yes	KPC2	YES	Positive	*K. pneumoniae*	*K. pneumoniae*	1	NO	N/A
ARLG-8000-P	Yes	KPC2	YES	Positive	*K. pneumoniae*	*K. pneumoniae*	1	NO	N/A
ARLG-7802-P	Yes	KPC3	NO	Negative	*K. pneumoniae*	*K. pneumoniae*	1	NO	N/A
ARLG-7616-P	Yes	KPC3	NO	Negative	*K. pneumoniae*	*K. pneumoniae*	1	NO	N/A
ARLG-8003-P	Yes	KPC2	YES	Positive	*K. pneumoniae*	*K. pneumoniae*	1	NO	N/A
ARLG-8004-P	Yes	KPC3	NO	Negative	*K. pneumoniae*	*K. pneumoniae*	1	NO	N/A
ARLG-7617-P	Yes	KPC2	YES	Positive	*K. pneumoniae*	*K. pneumoniae*	1	NO	N/A
ARLG-7963-P	No	N/A	NO	Negative	*K. pneumoniae*	*K. pneumoniae*	1	NO	N/A
ARLG-7620-P	Yes	KPC2	YES	Positive	*K. pneumoniae*	*K. pneumoniae*	1	NO	N/A
ARLG-7621-P	Yes	KPC3	NO	Negative	*K. pneumoniae*	*K. pneumoniae*	1	NO	N/A
ARLG-7901-P	Yes	KPC2	NO	Negative	*K. pneumoniae*	*K. pneumoniae*	1	NO	N/A
ARLG-7903-P	Yes	KPC3	NO	Negative	*K. pneumoniae*	*K. pneumoniae*	1	NO	N/A
ARLG-7622-P	Yes	KPC2	YES	Positive	*K. pneumoniae*	*K. pneumoniae*	1	NO	N/A
ARLG-7965-P	No	N/A	NO	Negative	*K. pneumoniae*	*K. pneumoniae*	1	NO	N/A
ARLG-7624-P	Yes	KPC2	YES	Positive	*K. pneumoniae*	*K. pneumoniae*	1	NO	N/A
ARLG-7688-P	Yes	KPC2	NO	Negative	*K. pneumoniae*	*K. pneumoniae*	1	NO	N/A

ARLG-7906-P	Yes	KPC2	NO	Negative	*K. pneumoniae*	*K. pneumoniae*	1	NO	N/A
ARLG-8007-P	Yes	KPC3	NO	Negative	*K. pneumoniae*	*K. pneumoniae*	1	NO	N/A
ARLG-8010-P	Yes	KPC31	NO	Negative	*K. pneumoniae*	*K. pneumoniae*	1	NO	N/A
ARLG-7775-P	Yes	KPC3	NO	Negative	*K. pneumoniae*	*K. pneumoniae*	1	NO	N/A
ARLG-7696-P	Yes	KPC2	YES	Positive	*K. pneumoniae*	*K. pneumoniae*	1	YES	1
ARLG-7697-P	Yes	KPC3	NO	Negative	*K. pneumoniae*	*K. pneumoniae*	1	NO	N/A
ARLG-7631-P	Yes	KPC2	YES	Positive	*K. pneumoniae*	*K. pneumoniae*	1	NO	N/A
ARLG-7791-P	Yes	KPC2	YES	Positive	*K. pneumoniae*	*K. pneumoniae*	1	NO	N/A
ARLG-7779-P	Yes	KPC2	YES	Positive	*K. pneumoniae*	*K. pneumoniae*	1	YES	1
ARLG-7642-P	Yes	KPC2	NO	Negative	*K. pneumoniae*	*K. pneumoniae*	1	NO	N/A
ARLG-7572-P	Yes	KPC3	NO	Negative	*K. pneumoniae*	*K. pneumoniae*	1	NO	N/A
ARLG-7646-P	Yes	KPC2	YES	Positive	*K. pneumoniae*	*K. pneumoniae*	1	NO	N/A
ARLG-7647-P	Yes	KPC3	NO	Negative	*K. pneumoniae*	*K. pneumoniae*	1	NO	N/A
ARLG-7654-P	Yes	KPC3	NO	Negative	*K. pneumoniae*	*K. pneumoniae*	1	NO	N/A
ARLG-7981-P	No	N/A	NO	Negative	*K. pneumoniae*	*K. pneumoniae*	1	NO	N/A
ARLG-7982-P	Yes	KPC3	NO	Negative	*K. pneumoniae*	*K. pneumoniae*	1	NO	N/A
ARLG-7656-P	Yes	KPC2	YES	Positive	*K. pneumoniae*	*K. pneumoniae*	1	NO	N/A
ARLG-8020-P	Yes	KPC2	YES	Positive	*K. pneumoniae*	*K. pneumoniae*	1	NO	N/A

ARLG-8021-P	Yes	KPC3	NO	Negative	*K. pneumoniae*	*K. pneumoniae*	1	NO	N/A
ARLG-7983-P	Yes	KPC3	NO	Negative	*K. pneumoniae*	*K. pneumoniae*	1	NO	N/A
ARLG-7673-P	Yes	KPC3	NO	Negative	*K. pneumoniae*	*K. pneumoniae*	1	NO	N/A
ARLG-7729-P	Yes	KPC2	NO	Negative	*K. pneumoniae*	*K. pneumoniae*	1	NO	N/A
ARLG-7735-P	Yes	KPC3	NO	Negative	*K. pneumoniae*	*K. pneumoniae*	1	NO	N/A
ARLG-7916-P	Yes	KPC3	NO	Negative	*K. pneumoniae*	*K. pneumoniae*	1	NO	N/A
ARLG-7996-U	Yes	KPC2	YES	Positive	*K. pneumoniae*	*K. pneumoniae*	1	NO	N/A
ARLG-7618-U	Yes	KPC2	YES	Positive	*K. pneumoniae*	*K. pneumoniae*	1	NO	N/A
ARLG-3495	Yes	KPC2	YES	Positive	*K. pneumoniae*	*K. pneumoniae*	1	NO	N/A
ARLG-3496	Yes	KPC3	NO	Negative	*K. pneumoniae*	*K. pneumoniae*	1	YES	4
ARLG-3499	Yes	KPC3	NO	Negative	*K. pneumoniae*	*K. pneumoniae*	1	NO	N/A
ARLG-3503	Yes	KPC3	NO	Negative	*K. pneumoniae*	*K. pneumoniae*	1	YES	4
ARLG-3504	Yes	KPC2	YES	Positive	*K. pneumoniae*	*K. pneumoniae*	1	NO	N/A
ARLG-3506	Yes	KPC2	NO	Negative	*K. pneumoniae*	*K. pneumoniae*	1	NO	N/A
ARLG-3508	Yes	KPC2	NO	Negative	*K. pneumoniae*	*K. pneumoniae*	1	NO	N/A
ARLG-3510	No	N/A	NO	Negative	*K. pneumoniae*	*K. pneumoniae*	1	YES	4
ARLG-3511	Yes	KPC3	YES	Positive	*K. pneumoniae*	*K. pneumoniae*	1	YES	3
ARLG-3517	Yes	KPC2	YES	Positive	*K. pneumoniae*	*K. pneumoniae*	1	NO	N/A
ARLG-3518	Yes	KPC2	YES	Positive	*K. pneumoniae*	*K. pneumoniae*	1	NO	N/A
ARLG-3520	Yes	KPC2	NO	Negative	*K. pneumoniae*	*K. pneumoniae*	1	NO	N/A
ARLG-3523	Yes	KPC3	NO	Negative	*K. pneumoniae*	*K. pneumoniae*	1	NO	N/A
ARLG-3524	No	N/A	NO	Negative	*K. pneumoniae*	*K. pneumoniae*	1	NO	N/A
ARLG-3527	Yes	KPC3	NO	Negative	*K. pneumoniae*	*K. pneumoniae*	1	NO	N/A
ARLG-3529	Yes	KPC2	YES	Positive	*K. pneumoniae*	*K. pneumoniae*	1	NO	N/A
ARLG-3530	Yes	KPC3	NO	Negative	*K. pneumoniae*	*K. pneumoniae*	1	NO	N/A

ARLG-3532	Yes	KPC3	NO	Negative	*K. pneumoniae*	*K. pneumoniae*	1	NO	N/A
ARLG-3533	No	N/A	NO	Negative	*K. pneumoniae*	*K. pneumoniae*	1	NO	N/A
ARLG-3534	Yes	KPC3	NO	Negative	*K. pneumoniae*	*K. pneumoniae*	1	NO	N/A
ARLG-3535	Yes	KPC3	NO	Negative	*K. pneumoniae*	*K. pneumoniae*	1	NO	N/A
ARLG-3536	No	N/A	NO	Negative	*K. pneumoniae*	*K. pneumoniae*	1	NO	N/A
ARLG-3546	Yes	KPC3	NO	Negative	*K. pneumoniae*	*K. pneumoniae*	1	NO	N/A
ARLG-3548	Yes	KPC3	NO	Negative	*K. pneumoniae*	*K. pneumoniae*	1	NO	N/A
ARLG-3553-P	Yes	KPC2	YES	Positive	*K. pneumoniae*	*K. pneumoniae*	1	NO	N/A
ARLG-3567-P	Yes	KPC2	YES	Positive	*K. pneumoniae*	*K. pneumoniae*	1	NO	N/A
ARLG-3575-P	Yes	KPC2	YES	Positive	*K. pneumoniae*	*K. pneumoniae*	1	NO	N/A
ARLG-3576-P	Yes	KPC2	YES	Positive	*K. pneumoniae*	*K. pneumoniae*	1	NO	N/A
ARLG-3578-P	Yes	KPC3	YES	Positive	*K. pneumoniae*	*K. pneumoniae*	1	NO	N/A
ARLG-3591-P	Yes	KPC3	NO	Negative	*K. pneumoniae*	*K. pneumoniae*	1	NO	N/A
ARLG-3594-P	Yes	KPC2	YES	Positive	*K. pneumoniae*	*K. pneumoniae*	1	NO	N/A
ARLG-3595-P	No	N/A	NO	Negative	*K. pneumoniae*	*K. pneumoniae*	1	NO	N/A
ARLG-3596-P	Yes	KPC2	YES	Positive	*K. pneumoniae*	*K. pneumoniae*	1	NO	N/A
ARLG-3598-P	Yes	KPC3	NO	Negative	*K. pneumoniae*	*K. pneumoniae*	1	NO	N/A
ARLG-3602-P	Yes	KPC2	NO	Negative	*K. pneumoniae*	*K. pneumoniae*	1	NO	N/A
ARLG-3603-P	Yes	KPC3	NO	Negative	*K. pneumoniae*	*K. pneumoniae*	1	NO	N/A
ARLG-3609-P	No	N/A	NO	Negative	*K. pneumoniae*	*K. pneumoniae*	1	NO	N/A
ARLG-3611-P	Yes	KPC3	NO	Negative	*K. pneumoniae*	*K. pneumoniae*	1	NO	N/A

ARLG-3612-P	Yes	KPC2	YES	Positive	*K. pneumoniae*	*K. pneumoniae*	1	NO	N/A
ARLG-3613-P	Yes	KPC3	NO	Negative	*K. pneumoniae*	*K. pneumoniae*	1	NO	N/A
ARLG-3614-P	Yes	KPC2	YES	Positive	*K. pneumoniae*	*K. pneumoniae*	1	NO	N/A
ARLG-3616-P	Yes	KPC2	NO	Negative	*K. pneumoniae*	*K. pneumoniae*	1	NO	N/A
ARLG-3617-P	Yes	KPC2	YES	Positive	*K. pneumoniae*	*K. pneumoniae*	1	NO	N/A
ARLG-3618-P	Yes	KPC3	NO	Negative	*K. pneumoniae*	*K. pneumoniae*	1	NO	N/A
ARLG-3621-P	Yes	KPC2	YES	Positive	*K. pneumoniae*	*K. pneumoniae*	1	NO	N/A
ARLG-3622-P	Yes	KPC3	YES	Positive	*K. pneumoniae*	*K. pneumoniae*	1	NO	N/A
ARLG-3623-P	Yes	KPC2	NO	Negative	*K. pneumoniae*	*K. pneumoniae*	1	NO	N/A
ARLG-3626-P	Yes	KPC2	YES	Positive	*K. pneumoniae*	*K. pneumoniae*	1	NO	N/A
ARLG-3627-P	No	N/A	NO	Negative	*K. pneumoniae*	*K. pneumoniae*	1	NO	N/A
ARLG-3628-P	Yes	KPC2	YES	Positive	*K. pneumoniae*	*K. pneumoniae*	1	NO	N/A
ARLG-3629-P	Yes	KPC2	NO	Negative	*K. pneumoniae*	*K. pneumoniae*	1	NO	N/A
ARLG-3630-P	Yes	KPC2	YES	Positive	*K. pneumoniae*	*K. pneumoniae*	1	NO	N/A
ARLG-3631-P	No	N/A	NO	Negative	*K. pneumoniae*	*K. pneumoniae*	1	NO	N/A
ARLG-3633-P	Yes	KPC2	NO	Negative	*K. pneumoniae*	*K. pneumoniae*	1	NO	N/A
ARLG-3634-P	Yes	KPC3	NO	Negative	*K. pneumoniae*	*K. pneumoniae*	1	NO	N/A
ARLG-3635-P	Yes	KPC2	NO	Negative	*K. pneumoniae*	*K. pneumoniae*	1	NO	N/A

ARLG-3637-P	Yes	KPC3	NO	Negative	*K. pneumoniae*	*K. pneumoniae*	1	NO	N/A
ARLG-3638-P	Yes	KPC2	NO	Negative	*K. pneumoniae*	*K. pneumoniae*	1	NO	N/A
ARLG-3639-P	No	N/A	NO	Negative	*K. pneumoniae*	*K. pneumoniae*	1	NO	N/A
ARLG-3640-P	No	N/A	NO	Negative	*K. pneumoniae*	*K. pneumoniae*	1	YES	3
ARLG-3641-P	Yes	KPC2	NO	Negative	*K. pneumoniae*	*K. pneumoniae*	1	NO	N/A
ARLG-3642-P	Yes	KPC2	NO	Negative	*K. pneumoniae*	*K. pneumoniae*	1	NO	N/A
ARLG-3643-P	Yes	KPC2	NO	Negative	*K. pneumoniae*	*K. pneumoniae*	1	NO	N/A
ARLG-3644-P	No	N/A	NO	Negative	*K. pneumoniae*	*K. pneumoniae*	1	NO	N/A
ARLG-3645-P	No	N/A	NO	Negative	*K. pneumoniae*	*K. pneumoniae*	1	NO	N/A
ARLG-3646-P	No	N/A	NO	Negative	*K. pneumoniae*	*K. pneumoniae*	1	YES	4
ARLG-4550-P	Yes	KPC3	NO	Negative	*K. pneumoniae*	*K. pneumoniae*	1	NO	N/A
ARLG-4541-P	Yes	KPC3	NO	Negative	*K. pneumoniae*	*K. pneumoniae*	1	NO	N/A
ARLG-4542-P	Yes	KPC2	NO	Negative	*K. pneumoniae*	*K. pneumoniae*	1	NO	N/A
ARLG-4552-P	Yes	KPC3	YES	Positive	*K. pneumoniae*	*K. pneumoniae*	1	NO	N/A
ARLG-4551-P	Yes	KPC2	YES	Positive	*K. pneumoniae*	*K. pneumoniae*	1	NO	N/A
ARLG-4543-P	No	N/A	NO	Negative	*K. pneumoniae*	*K. pneumoniae*	1	NO	N/A
ARLG-4289-P	Yes	KPC2	YES	Positive	*K. pneumoniae*	*K. pneumoniae*	1	YES	2
ARLG-4546-P	Yes	KPC2	NO	Negative	*K. pneumoniae*	*K. pneumoniae*	1	NO	N/A

ARLG-4544-P	Yes	KPC3	YES	Positive	*K. pneumoniae*	*K. pneumoniae*	1	YES	2
ARLG-4534-P	Yes	KPC2	YES	Positive	*K. pneumoniae*	*K. pneumoniae*	1	NO	N/A
ARLG-4537-P	Yes	KPC3	NO	Negative	*K. pneumoniae*	*K. pneumoniae*	1	NO	N/A
ARLG-4304-P	No	N/A	NO	Negative	*K. pneumoniae*	*K. pneumoniae*	1	NO	N/A
ARLG-4228-P	Yes	KPC3	NO	Negative	*K. pneumoniae*	*K. pneumoniae*	1	NO	N/A
ARLG-4229-P	Yes	KPC3	NO	Negative	*K. pneumoniae*	*K. pneumoniae*	1	NO	N/A
ARLG-4232	Yes	KPC3	YES	Positive	*K. pneumoniae*	*K. pneumoniae*	1	YES	2
ARLG-4234	Yes	KPC2	YES	Positive	*K. pneumoniae*	*K. pneumoniae*	1	NO	N/A
ARLG-4235-P	Yes	KPC2	YES	Positive	*K. pneumoniae*	*K. pneumoniae*	1	NO	N/A
ARLG-4236-P	Yes	KPC2	YES	Positive	*K. pneumoniae*	*K. pneumoniae*	1	NO	N/A
ARLG-4237	No	N/A	NO	Negative	*K. pneumoniae*	*K. pneumoniae*	1	NO	N/A
ARLG-4241	Yes	KPC2	YES	Positive	*K. pneumoniae*	*K. pneumoniae*	1	NO	N/A
ARLG-4242	Yes	KPC2	YES	Positive	*K. pneumoniae*	*K. pneumoniae*	1	YES	2
ARLG-4243	Yes	KPC2	NO	Negative	*K. pneumoniae*	*K. pneumoniae*	1	NO	N/A
ARLG-4244	No	N/A	NO	Negative	*K. pneumoniae*	*K. pneumoniae*	1	NO	N/A
ARLG-4245	Yes	KPC2	YES	Positive	*K. pneumoniae*	*K. pneumoniae*	1	NO	N/A
ARLG-4246	Yes	KPC2	YES	Positive	*K. pneumoniae*	*K. pneumoniae*	1	YES	1
ARLG-4249	Yes	KPC3	NO	Negative	*K. pneumoniae*	*K. pneumoniae*	1	NO	N/A
ARLG-4250	Yes	KPC2	YES	Positive	*K. pneumoniae*	*K. pneumoniae*	1	NO	N/A
ARLG-4251	No	N/A	NO	Negative	*K. pneumoniae*	*K. pneumoniae*	1	NO	N/A
ARLG-4252	Yes	KPC3	NO	Negative	*K. pneumoniae*	*K. pneumoniae*	1	NO	N/A
ARLG-4257	Yes	KPC2	YES	Positive	*K. pneumoniae*	*K. pneumoniae*	1	NO	N/A
ARLG-4258	Yes	KPC3	YES	Positive	*K. pneumoniae*	*K. pneumoniae*	1	NO	N/A
ARLG-4260	Yes	KPC2	NO	Negative	*K. pneumoniae*	*K. pneumoniae*	1	NO	N/A
ARLG-4261	No	N/A	NO	Negative	*K. pneumoniae*	*K. pneumoniae*	1	NO	N/A

ARLG-4262	No	N/A	NO	Negative	*K. pneumoniae*	*K. pneumoniae*	1	NO	N/A
ARLG-4263	No	N/A	NO	Negative	*K. pneumoniae*	*K. pneumoniae*	1	NO	N/A
ARLG-4266	Yes	KPC2	NO	Negative	*K. pneumoniae*	*K. pneumoniae*	1	NO	N/A
ARLG-4374	Yes	KPC2	YES	Positive	*K. pneumoniae*	*K. pneumoniae*	1	NO	N/A
ARLG-4387	Yes	KPC2	YES	Positive	*K. pneumoniae*	*K. pneumoniae*	1	NO	N/A
ARLG-4390	Yes	KPC3	NO	Negative	*K. pneumoniae*	*K. pneumoniae*	1	NO	N/A
ARLG-4391	No	N/A	NO	Negative	*K. pneumoniae*	*K. pneumoniae*	1	NO	N/A
ARLG-4354	Yes	KPC2	YES	Positive	*K. pneumoniae*	*K. pneumoniae*	1	NO	N/A
ARLG-4356	No	N/A	NO	Negative	*K. pneumoniae*	*K. pneumoniae*	1	NO	N/A
ARLG-4469	Yes	KPC2	YES	Positive	*K. pneumoniae*	*K. pneumoniae*	1	NO	N/A
ARLG-4474	Yes	KPC3	NO	Negative	*K. pneumoniae*	*K. pneumoniae*	1	NO	N/A
ARLG-4476	Yes	KPC3	NO	Negative	*K. pneumoniae*	*K. pneumoniae*	1	NO	N/A
ARLG-4433	Yes	KPC2	YES	Positive	*K. pneumoniae*	*K. pneumoniae*	1	NO	N/A
ARLG-4434	Yes	KPC2	YES	Positive	*K. pneumoniae*	*K. pneumoniae*	1	NO	N/A
ARLG-4435	Yes	KPC3	NO	Negative	*K. pneumoniae*	*K. pneumoniae*	1	NO	N/A
ARLG-4440	Yes	KPC3	YES	Positive	*K. pneumoniae*	*K. pneumoniae*	1	NO	N/A
ARLG-4441	Yes	KPC2	YES	Positive	*K. pneumoniae*	*K. pneumoniae*	1	NO	N/A
ARLG-4267	Yes	KPC2	YES	Positive	*K. pneumoniae*	*K. pneumoniae*	1	NO	N/A
ARLG-4268	Yes	KPC2	YES	Positive	*K. pneumoniae*	*K. pneumoniae*	1	NO	N/A
ARLG-4269	Yes	KPC2	YES	Positive	*K. pneumoniae*	*K. pneumoniae*	1	NO	N/A
ARLG-4272	No	N/A	NO	Negative	*K. pneumoniae*	*K. pneumoniae*	1	NO	N/A
ARLG-4273	Yes	KPC2	YES	Positive	*K. pneumoniae*	*K. pneumoniae*	1	NO	N/A
ARLG-4274	No	N/A	NO	Negative	*K. pneumoniae*	*K. pneumoniae*	1	NO	N/A
ARLG-4184	Yes	KPC3	NO	Negative	*K. pneumoniae*	*K. pneumoniae*	1	NO	N/A
ARLG-4185	Yes	KPC3	NO	Negative	*K. pneumoniae*	*K. pneumoniae*	1	NO	N/A
ARLG-4187	Yes	KPC2	YES	Positive	*K. pneumoniae*	*K. pneumoniae*	1	NO	N/A
ARLG-4190	Yes	KPC3	YES	Positive	*K. pneumoniae*	*K. pneumoniae*	1	YES	3
ARLG-4193	Yes	KPC2	YES	Negative	*K. pneumoniae*	*K. pneumoniae*	1	NO	N/A
ARLG-4195	No	N/A	NO	Negative	*K. pneumoniae*	*K. pneumoniae*	1	NO	N/A
ARLG-4196	No	N/A	NO	Negative	*K. pneumoniae*	*K. pneumoniae*	1	NO	N/A

ARLG-4197	No	N/A	NO	Negative	*K. pneumoniae*	*K. pneumoniae*	1	NO	N/A
ARLG-4198	Yes	KPC2	YES	Positive	*K. pneumoniae*	*K. pneumoniae*	1	NO	N/A
ARLG-4199	Yes	KPC2	YES	Positive	*K. pneumoniae*	*K. pneumoniae*	1	NO	N/A
ARLG-4557	Yes	KPC3	YES	Negative	*K. pneumoniae*	*K. pneumoniae*	1	NO	N/A
ARLG-4558	Yes	KPC3	YES	Positive	*K. pneumoniae*	*K. pneumoniae*	1	YES	2
ARLG-4554	Yes	KPC3	NO	Negative	*K. pneumoniae*	*K. pneumoniae*	1	YES	3
ARLG-4555	Yes	KPC2	NO	Negative	*K. pneumoniae*	*K. pneumoniae*	1	NO	N/A
ARLG-4556	Yes	KPC3	NO	Negative	*K. pneumoniae*	*K. pneumoniae*	1	NO	N/A
ARLG-4378	Yes	KPC2	YES	Positive	*K. pneumoniae*	*K. pneumoniae*	1	NO	N/A
ARLG-4380	Yes	KPC3	NO	Negative	*K. pneumoniae*	*K. pneumoniae*	1	NO	N/A
ARLG-4382	Yes	KPC2	NO	Negative	*K. pneumoniae*	*K. pneumoniae*	1	NO	N/A
ARLG-4358	Yes	KPC2	YES	Positive	*K. pneumoniae*	*K. pneumoniae*	1	NO	N/A
ARLG-4359	No	N/A	NO	Negative	*K. pneumoniae*	*K. pneumoniae*	1	NO	N/A
ARLG-4360	Yes	KPC2	NO	Negative	*K. pneumoniae*	*K. pneumoniae*	1	NO	N/A
ARLG-4364	Yes	KPC3	NO	Negative	*K. pneumoniae*	*K. pneumoniae*	1	NO	N/A
ARLG-4365	Yes	KPC2	NO	Negative	*K. pneumoniae*	*K. pneumoniae*	1	NO	N/A
ARLG-4506	No	N/A	NO	Negative	*K. pneumoniae*	*K. pneumoniae*	1	NO	N/A
ARLG-4507	Yes	KPC2	NO	Negative	*K. pneumoniae*	*K. pneumoniae*	1	NO	N/A
ARLG-4508	Yes	KPC3	NO	Negative	*K. pneumoniae*	*K. pneumoniae*	1	NO	N/A
ARLG-4509	Yes	KPC3	NO	Negative	*K. pneumoniae*	*K. pneumoniae*	1	NO	N/A
ARLG-4510	Yes	KPC3	NO	Negative	*K. pneumoniae*	*K. pneumoniae*	1	NO	N/A
ARLG-4511	Yes	KPC3	NO	Negative	*K. pneumoniae*	*K. pneumoniae*	1	NO	N/A
ARLG-4492	Yes	KPC3	NO	Negative	*K. pneumoniae*	*K. pneumoniae*	1	NO	N/A
ARLG-4493	Yes	KPC3	NO	Negative	*K. pneumoniae*	*K. pneumoniae*	1	NO	N/A
ARLG-4495	Yes	KPC3	NO	Negative	*K. pneumoniae*	*K. pneumoniae*	1	NO	N/A
ARLG-4496	Yes	KPC2	YES	Positive	*K. pneumoniae*	*K. pneumoniae*	1	NO	N/A
ARLG-4497	Yes	KPC3	YES	Positive	*K. pneumoniae*	*K. pneumoniae*	1	YES	2
ARLG-4498	Yes	KPC3	YES	Positive	*K. pneumoniae*	*K. pneumoniae*	1	NO	N/A
ARLG-4499	No	N/A	NO	Negative	*K. pneumoniae*	*K. pneumoniae*	1	NO	N/A
ARLG-4501	Yes	KPC3	NO	Negative	*K. pneumoniae*	*K. pneumoniae*	1	NO	N/A

ARLG-4502	Yes	KPC2	YES	Positive	*K. pneumoniae*	*K. pneumoniae*	1	NO	N/A
ARLG-4503	Yes	KPC3	NO	Negative	*K. pneumoniae*	*K. pneumoniae*	1	NO	N/A
ARLG-4445	No	N/A	NO	Negative	*K. pneumoniae*	*K. pneumoniae*	1	NO	N/A
ARLG-4446	Yes	KPC2	YES	Positive	*K. pneumoniae*	*K. pneumoniae*	1	NO	N/A
ARLG-4449	Yes	KPC2	YES	Positive	*K. pneumoniae*	*K. pneumoniae*	1	NO	N/A
ARLG-4450	No	N/A	NO	Negative	*K. pneumoniae*	*K. pneumoniae*	1	NO	N/A
ARLG-4451	Yes	KPC2	YES	Positive	*K. pneumoniae*	*K. pneumoniae*	1	NO	N/A
ARLG-4452	Yes	KPC3	NO	Negative	*K. pneumoniae*	*K. pneumoniae*	1	NO	N/A
ARLG-4454	Yes	KPC2	YES	Positive	*K. pneumoniae*	*K. pneumoniae*	1	NO	N/A
ARLG-4456	Yes	KPC2	YES	Positive	*K. pneumoniae*	*K. pneumoniae*	1	NO	N/A
ARLG-4457	Yes	KPC3	NO	Negative	*K. pneumoniae*	*K. pneumoniae*	1	NO	N/A
ARLG-4459	Yes	KPC3	NO	Negative	*K. pneumoniae*	*K. pneumoniae*	1	NO	N/A
ARLG-4461	Yes	KPC2	YES	Positive	*K. pneumoniae*	*K. pneumoniae*	1	NO	N/A
ARLG-4513	No	N/A	NO	Negative	*K. pneumoniae*	*K. pneumoniae*	1	NO	N/A
ARLG-4514	Yes	KPC3	YES	Positive	*K. pneumoniae*	*K. pneumoniae*	1	YES	2
ARLG-4517	Yes	KPC3	NO	Negative	*K. pneumoniae*	*K. pneumoniae*	1	NO	N/A
ARLG-4521	Yes	KPC3	NO	Negative	*K. pneumoniae*	*K. pneumoniae*	1	NO	N/A
ARLG-4524	Yes	KPC3	NO	Negative	*K. pneumoniae*	*K. pneumoniae*	1	NO	N/A
ARLG-4532	Yes	KPC3	NO	Negative	*K. pneumoniae*	*K. pneumoniae*	1	NO	N/A
ARLG-4529	Yes	KPC2	YES	Positive	*K. pneumoniae*	*K. pneumoniae*	1	NO	N/A
ARLG-4527	Yes	KPC2	YES	Positive	*K. pneumoniae*	*K. pneumoniae*	1	NO	N/A
ARLG-4530	Yes	KPC3	NO	Negative	*K. pneumoniae*	*K. pneumoniae*	1	NO	N/A
ARLG-4528	Yes	KPC3	NO	Negative	*K. pneumoniae*	*K. pneumoniae*	1	NO	N/A
ARLG-4206	No	N/A	NO	Negative	*K. pneumoniae*	*K. pneumoniae*	1	NO	N/A
ARLG-4208	Yes	KPC2	YES	Positive	*K. pneumoniae*	*K. pneumoniae*	1	NO	N/A
ARLG-4211	No	N/A	NO	Negative	*K. pneumoniae*	*K. pneumoniae*	1	NO	N/A
ARLG-4212	Yes	KPC3	NO	Negative	*K. pneumoniae*	*K. pneumoniae*	1	NO	N/A
ARLG-4213	Yes	KPC2	YES	Positive	*K. pneumoniae*	*K. pneumoniae*	1	NO	N/A
ARLG-4214	No	N/A	NO	Negative	*K. pneumoniae*	*K. pneumoniae*	1	NO	N/A
ARLG-4215	No	N/A	NO	Negative	*K. pneumoniae*	*K. pneumoniae*	1	NO	N/A

ARLG-4219	Yes	KPC3	NO	Negative	*K. pneumoniae*	*K. pneumoniae*	1	NO	N/A
ARLG-4220	Yes	KPC2	NO	Negative	*K. pneumoniae*	*K. pneumoniae*	1	NO	N/A
ARLG-4222	Yes	KPC3	NO	Negative	*K. pneumoniae*	*K. pneumoniae*	1	NO	N/A
ARLG-4370	No	N/A	NO	Negative	*K. pneumoniae*	*K. pneumoniae*	1	NO	N/A
ARLG-4371	Yes	KPC3	NO	Negative	*K. pneumoniae*	*K. pneumoniae*	1	NO	N/A
ARLG-3302-P	Yes	KPC2	YES	Positive	*K. pneumoniae*	*K. pneumoniae*	1	NO	N/A
ARLG-3309-P	Yes	KPC2	YES	Positive	*K. pneumoniae*	*K. pneumoniae*	1	NO	N/A
ARLG-3316-P	No	N/A	NO	Negative	*K. pneumoniae*	*K. pneumoniae*	1	NO	N/A
ARLG-3318-P	Yes	KPC2	YES	Positive	*K. pneumoniae*	*K. pneumoniae*	1	NO	N/A
ARLG-3327-P	No	N/A	NO	Negative	*K. pneumoniae*	*K. pneumoniae*	1	NO	N/A
ARLG-3336-P	Yes	KPC3	NO	Negative	*K. pneumoniae*	*K. pneumoniae*	1	NO	N/A
ARLG-3341-P	Yes	KPC3	NO	Negative	*K. pneumoniae*	*K. pneumoniae*	1	YES	4
ARLG-3345-P	Yes	KPC3	NO	Negative	*K. pneumoniae*	*K. pneumoniae*	1	NO	N/A
ARLG-3356-P	Yes	KPC3	NO	Negative	*K. pneumoniae*	*K. pneumoniae*	1	NO	N/A
ARLG-3360-P	Yes	KPC3	NO	Negative	*K. pneumoniae*	*K. pneumoniae*	1	NO	N/A
ARLG-3363-P	Yes	KPC3	NO	Negative	*K. pneumoniae*	*K. pneumoniae*	1	NO	N/A
ARLG-3366-P	Yes	KPC3	NO	Negative	*K. pneumoniae*	*K. pneumoniae*	1	NO	N/A
ARLG-3372-P	Yes	KPC3	NO	Negative	*K. pneumoniae*	*K. pneumoniae*	1	NO	N/A
ARLG-3380-P	Yes	KPC2	YES	Positive	*K. pneumoniae*	*K. pneumoniae*	1	NO	N/A
ARLG-3414-P	Yes	KPC3	NO	Negative	*K. pneumoniae*	*K. pneumoniae*	1	NO	N/A

ARLG-3415-P	Yes	KPC3	NO	Negative	*K. pneumoniae*	*K. pneumoniae*	1	NO	N/A
ARLG-3451-P	No	N/A	NO	Negative	*K. pneumoniae*	*K. pneumoniae*	1	NO	N/A
ARLG-3463-P	Yes	KPC2	YES	Positive	*K. pneumoniae*	*K. pneumoniae*	1	NO	N/A
ARLG-3467-P	No	N/A	NO	Negative	*K. pneumoniae*	*K. pneumoniae*	1	NO	N/A
ARLG-3473-P	No	N/A	NO	Negative	*K. pneumoniae*	*K. pneumoniae*	1	NO	N/A
ARLG-3484-P	No	N/A	NO	Negative	*K. pneumoniae*	*K. pneumoniae*	1	YES	3
ARLG-3489-P	Yes	KPC3	NO	Negative	*K. pneumoniae*	*K. pneumoniae*	1	NO	N/A
ARLG-4419-P	Yes	KPC2	YES	Positive	*K. pneumoniae*	*K. pneumoniae*	1	NO	N/A
ARLG-4420-P	Yes	KPC2	NO	Negative	*K. pneumoniae*	*K. pneumoniae*	1	NO	N/A
ARLG-4421-P	Yes	KPC2	YES	Positive	*K. pneumoniae*	*K. pneumoniae*	1	NO	N/A
ARLG-4422-P	Yes	KPC3	NO	Negative	*K. pneumoniae*	*K. pneumoniae*	1	NO	N/A
ARLG-4423-P	Yes	KPC2	NO	Negative	*K. pneumoniae*	*K. pneumoniae*	1	NO	N/A
ARLG-4424-P	Yes	KPC2	YES	Positive	*K. pneumoniae*	*K. pneumoniae*	1	NO	N/A
ARLG-4425-P	Yes	KPC3	NO	Negative	*K. pneumoniae*	*K. pneumoniae*	1	NO	N/A
ARLG-4314-P	Yes	KPC3	NO	Negative	*K. pneumoniae*	*K. pneumoniae*	1	NO	N/A
ARLG-4300-P	Yes	KPC2	YES	Positive	*K. pneumoniae*	*K. pneumoniae*	1	NO	N/A
ARLG-4301-P	Yes	KPC3	NO	Negative	*K. pneumoniae*	*K. pneumoniae*	1	NO	N/A
ARLG-4305-P	No	N/A	NO	Negative	*K. pneumoniae*	*K. pneumoniae*	1	YES	3

ARLG-4336-P	Yes	KPC3	NO	Negative	*K. pneumoniae*	*K. pneumoniae*	1	NO	N/A
ARLG-4340-P	Yes	KPC8	NO	Negative	*K. pneumoniae*	*K. pneumoniae*	1	NO	N/A
ARLG-4173-P	No	N/A	NO	Negative	*K. pneumoniae*	*K. pneumoniae*	1	NO	N/A
ARLG-4384-P	No	N/A	NO	Negative	*K. pneumoniae*	*K. pneumoniae*	1	NO	N/A
ARLG-4279-P	Yes	KPC3	NO	Negative	*K. pneumoniae*	*K. pneumoniae*	1	NO	N/A
ARLG-4480-P	Yes	KPC3	NO	Negative	*K. pneumoniae*	*K. pneumoniae*	1	NO	N/A
ARLG-4481-P	Yes	KPC3	NO	Negative	*K. pneumoniae*	*K. pneumoniae*	1	NO	N/A
ARLG-4160-P	Yes	KPC2	YES	Positive	*K. pneumoniae*	*K. pneumoniae*	1	NO	N/A
ARLG-4161-P	Yes	KPC2	YES	Positive	*K. pneumoniae*	*K. pneumoniae*	1	NO	N/A
ARLG-4162-P	Yes	KPC2	YES	Positive	*K. pneumoniae*	*K. pneumoniae*	1	YES	2
ARLG-4176-P	No	N/A	NO	Negative	*K. pneumoniae*	*K. pneumoniae*	1	YES	3
ARLG-4322-P	Yes	KPC3	NO	Negative	*K. pneumoniae*	*K. pneumoniae*	1	NO	N/A
ARLG-4323-P	Yes	KPC2	YES	Positive	*K. pneumoniae*	*K. pneumoniae*	1	YES	1
ARLG-4328-P	Yes	KPC3	NO	Negative	*K. pneumoniae*	*K. pneumoniae*	1	NO	N/A
ARLG-4308-P	Yes	KPC3	NO	Negative	*K. pneumoniae*	*K. pneumoniae*	1	NO	N/A
ARLG-4292-P	Yes	KPC2	YES	Positive	*K. pneumoniae*	*K. pneumoniae*	1	NO	N/A
ARLG-4293-P	Yes	KPC2	NO	Negative	*K. pneumoniae*	*K. pneumoniae*	1	NO	N/A
ARLG-4294-P	Yes	KPC3	NO	Negative	*K. pneumoniae*	*K. pneumoniae*	1	NO	N/A

ARLG-4426-P	Yes	KPC2	YES	Positive	*K. pneumoniae*	*K. pneumoniae*	1	NO	N/A
ARLG-4427-P	Yes	KPC3	NO	Negative	*K. pneumoniae*	*K. pneumoniae*	1	NO	N/A
ARLG-4428-P	Yes	KPC2	YES	Positive	*K. pneumoniae*	*K. pneumoniae*	1	NO	N/A
ARLG-4429-P	Yes	KPC2	YES	Positive	*K. pneumoniae*	*K. pneumoniae*	1	NO	N/A
ARLG-4411-P	Yes	KPC2	YES	Positive	*K. pneumoniae*	*K. pneumoniae*	1	NO	N/A
ARLG-4332-P	Yes	KPC3	NO	Negative	*K. pneumoniae*	*K. pneumoniae*	1	NO	N/A
ARLG-4181-P	Yes	KPC3	NO	Negative	*K. pneumoniae*	*K. pneumoniae*	1	NO	N/A
ARLG-4342-P	Yes	KPC3	NO	Negative	*K. pneumoniae*	*K. pneumoniae*	1	NO	N/A
ARLG-4179-P	No	N/A	NO	Negative	*K. pneumoniae*	*K. pneumoniae*	1	NO	N/A
ARLG-4484-P	Yes	KPC2	NO	Negative	*K. pneumoniae*	*K. pneumoniae*	1	NO	N/A
ARLG-4487-P	Yes	KPC3	NO	Negative	*K. pneumoniae*	*K. pneumoniae*	1	NO	N/A
ARLG-4488-P	Yes	KPC2	NO	Negative	*K. pneumoniae*	*K. pneumoniae*	1	NO	N/A
ARLG-4296-P	Yes	KPC3	NO	Negative	*K. pneumoniae*	*K. pneumoniae*	1	NO	N/A
ARLG-4297-P	Yes	KPC3	NO	Negative	*K. pneumoniae*	*K. pneumoniae*	1	NO	N/A
ARLG-4344-P	Yes	KPC2	YES	Positive	*K. pneumoniae*	*K. pneumoniae*	1	NO	N/A
ARLG-4345-P	Yes	KPC3	NO	Negative	*K. pneumoniae*	*K. pneumoniae*	1	NO	N/A
ARLG-4283-P	Yes	KPC3	NO	Negative	*K. pneumoniae*	*K. pneumoniae*	1	NO	N/A
ARLG-4287-P	Yes	KPC2	NO	Negative	*K. pneumoniae*	*K. pneumoniae*	1	NO	N/A

ARLG-4275-P	Yes	KPC2	YES	Positive	*K. pneumoniae*	*K. pneumoniae*	1	NO	N/A
ARLG-4330-P	No	N/A	NO	Negative	*K. pneumoniae*	*K. pneumoniae*	1	NO	N/A
ARLG-4333-P	Yes	KPC3	NO	Negative	*K. pneumoniae*	*K. pneumoniae*	1	NO	N/A
ARLG-4396-P	No	N/A	NO	Negative	*K. pneumoniae*	*K. pneumoniae*	1	NO	N/A
ARLG-4397-P	Yes	KPC3	NO	Negative	*K. pneumoniae*	*K. pneumoniae*	1	NO	N/A
ARLG-4430-P	Yes	KPC2	YES	Positive	*K. pneumoniae*	*K. pneumoniae*	1	NO	N/A
ARLG-4431-P	Yes	KPC2	YES	Positive	*K. pneumoniae*	*K. pneumoniae*	1	NO	N/A
ARLG-4412-P	Yes	KPC3	NO	Negative	*K. pneumoniae*	*K. pneumoniae*	1	NO	N/A
ARLG-4413-P	No	N/A	NO	Negative	*K. pneumoniae*	*K. pneumoniae*	1	NO	N/A
ARLG-4416-P	Yes	KPC2	YES	Positive	*K. pneumoniae*	*K. pneumoniae*	1	NO	N/A
ARLG-4334-P	Yes	KPC3	NO	Negative	*K. pneumoniae*	*K. pneumoniae*	1	NO	N/A
ARLG-4483-P	Yes	KPC2	NO	Negative	*K. pneumoniae*	*K. pneumoniae*	1	NO	N/A
ARLG-4489-P	Yes	KPC2	NO	Negative	*K. pneumoniae*	*K. pneumoniae*	1	NO	N/A
ARLG-4490-P	Yes	KPC2	NO	Negative	*K. pneumoniae*	*K. pneumoniae*	1	NO	N/A
ARLG-4491-P	Yes	KPC2	NO	Negative	*K. pneumoniae*	*K. pneumoniae*	1	NO	N/A
ARLG-4324-P	Yes	KPC3	NO	Negative	*K. pneumoniae*	*K. pneumoniae*	1	NO	N/A
ARLG-4325-P	No	N/A	NO	Negative	*K. pneumoniae*	*K. pneumoniae*	1	NO	N/A
ARLG-4225-P	No	N/A	NO	Negative	*K. pneumoniae*	*K. pneumoniae*	1	NO	N/A

ARLG-4317-P	Yes	KPC2	NO	Negative	*K. pneumoniae*	*K. pneumoniae*	1	NO	N/A
ARLG-4462-P	Yes	KPC2	YES	Positive	*K. pneumoniae*	*K. pneumoniae*	1	NO	N/A
ARLG-4319-P	Yes	KPC2	NO	Negative	*K. pneumoniae*	*K. pneumoniae*	1	NO	N/A
ARLG-4320-P	No	N/A	NO	Negative	*K. pneumoniae*	*K. pneumoniae*	1	NO	N/A
ARLG-4338-P	Yes	KPC2	YES	Positive	*K. pneumoniae*	*K. pneumoniae*	1	NO	N/A
ARLG-4339-P	Yes	KPC3	NO	Negative	*K. pneumoniae*	*K. pneumoniae*	1	NO	N/A
ARLG-4278-P	Yes	KPC2	YES	Positive	*K. pneumoniae*	*K. pneumoniae*	1	NO	N/A
ARLG-4399-P	Yes	KPC2	NO	Negative	*K. pneumoniae*	*K. pneumoniae*	1	NO	N/A
ARLG-4230-P	Yes	KPC2	NO	Negative	*K. pneumoniae*	*K. pneumoniae*	1	NO	N/A
ARLG-4400-P	Yes	KPC2	NO	Negative	*K. pneumoniae*	*K. pneumoniae*	1	NO	N/A
ARLG-4403-P	Yes	KPC2	NO	Negative	*K. pneumoniae*	*K. pneumoniae*	1	NO	N/A
ARLG-4404-P	Yes	KPC2	NO	Negative	*K. pneumoniae*	*K. pneumoniae*	1	NO	N/A
ARLG-4408-P	No	N/A	NO	Negative	*K. pneumoniae*	*K. pneumoniae*	1	NO	N/A
ARLG-4392-P	No	N/A	NO	Negative	*K. pneumoniae*	*K. pneumoniae*	1	NO	N/A
ARLG-4169-P	Yes	KPC3	NO	Negative	*K. pneumoniae*	*K. pneumoniae*	1	NO	N/A
ARLG-4398-P	Yes	KPC3	NO	Negative	*K. pneumoniae*	*K. pneumoniae*	1	NO	N/A
ARLG-4172-P	No	N/A	NO	Negative	*K. pneumoniae*	*K. pneumoniae*	1	NO	N/A
ARLG-4155-P	Yes	KPC2	YES	Positive	*K. pneumoniae*	*K. pneumoniae*	1	NO	N/A

ARLG-4156-P	Yes	KPC2	YES	Positive	*K. pneumoniae*	*K. pneumoniae*	1	NO	N/A
ARLG-4157-P	Yes	KPC2	NO	Negative	*K. pneumoniae*	*K. pneumoniae*	1	NO	N/A
ARLG-3177	Yes	KPC2	YES	Positive	*K. pneumoniae*	*K. pneumoniae*	1	NO	N/A
ARLG-3179	Yes	KPC2	YES	Positive	*K. pneumoniae*	*K. pneumoniae*	1	NO	N/A
ARLG-3180	Yes	KPC2	YES	Positive	*K. pneumoniae*	*K. pneumoniae*	1	NO	N/A
ARLG-3181	Yes	KPC2	YES	Positive	*K. pneumoniae*	*K. pneumoniae*	1	NO	N/A
ARLG-3182	Yes	KPC2	NO	Negative	*K. pneumoniae*	*K. pneumoniae*	1	NO	N/A
ARLG-3183	Yes	KPC2	YES	Positive	*K. pneumoniae*	*K. pneumoniae*	1	NO	N/A
ARLG-3185	No	N/A	NO	Negative	*K. pneumoniae*	*K. pneumoniae*	1	NO	N/A
ARLG-3186	No	N/A	NO	Negative	*K. pneumoniae*	*K. pneumoniae*	1	NO	N/A
ARLG-3187	Yes	KPC2	NO	Negative	*K. pneumoniae*	*K. pneumoniae*	1	NO	N/A
ARLG-3188	Yes	KPC2	YES	Positive	*K. pneumoniae*	*K. pneumoniae*	1	NO	N/A
ARLG-3192	No	N/A	NO	Negative	*K. pneumoniae*	*K. pneumoniae*	1	NO	N/A
ARLG-3194	Yes	KPC2	NO	Negative	*K. pneumoniae*	*K. pneumoniae*	1	NO	N/A
ARLG-3195	Yes	KPC2	YES	Positive	*K. pneumoniae*	*K. pneumoniae*	1	NO	N/A
ARLG-3196	Yes	KPC2	YES	Positive	*K. pneumoniae*	*K. pneumoniae*	1	NO	N/A
ARLG-3197	Yes	KPC2	YES	Positive	*K. pneumoniae*	*K. pneumoniae*	1	NO	N/A
ARLG-3198	Yes	KPC2	NO	Negative	*K. pneumoniae*	*K. pneumoniae*	1	NO	N/A
ARLG-3199	Yes	KPC2	NO	Negative	*K. pneumoniae*	*K. pneumoniae*	1	NO	N/A
ARLG-3204	Yes	KPC2	YES	Positive	*K. pneumoniae*	*K. pneumoniae*	1	NO	N/A
ARLG-3205	Yes	KPC2	YES	Positive	*K. pneumoniae*	*K. pneumoniae*	1	NO	N/A
ARLG-3206	Yes	KPC2	YES	Positive	*K. pneumoniae*	*K. pneumoniae*	1	NO	N/A
ARLG-3208	Yes	KPC2	YES	Positive	*K. pneumoniae*	*K. pneumoniae*	1	NO	N/A
ARLG-3211	Yes	KPC2	YES	Positive	*K. pneumoniae*	*K. pneumoniae*	1	NO	N/A
ARLG-3213	Yes	KPC2	YES	Positive	*K. pneumoniae*	*K. pneumoniae*	1	NO	N/A
ARLG-3214	Yes	KPC2	YES	Positive	*K. pneumoniae*	*K. pneumoniae*	1	NO	N/A
ARLG-3215	Yes	KPC2	YES	Positive	*K. pneumoniae*	*K. pneumoniae*	1	NO	N/A
ARLG-3218	Yes	KPC2	NO	Negative	*K. pneumoniae*	*K. pneumoniae*	1	NO	N/A

ARLG-3220	Yes	KPC2	YES	Positive	*K. pneumoniae*	*K. pneumoniae*	1	NO	N/A
ARLG-3222	Yes	KPC2	YES	Positive	*K. pneumoniae*	*K. pneumoniae*	1	NO	N/A
ARLG-3223	Yes	KPC2	YES	Positive	*K. pneumoniae*	*K. pneumoniae*	1	NO	N/A
ARLG-3224	Yes	KPC2	YES	Positive	*K. pneumoniae*	*K. pneumoniae*	1	YES	1
ARLG-3226	Yes	KPC3	YES	Positive	*K. pneumoniae*	*K. pneumoniae*	1	NO	N/A
ARLG-3234	Yes	KPC2	YES	Positive	*K. pneumoniae*	*K. pneumoniae*	1	NO	N/A
ARLG-3235	Yes	KPC2	YES	Positive	*K. pneumoniae*	*K. pneumoniae*	1	NO	N/A
ARLG-3237	Yes	KPC2	YES	Positive	*K. pneumoniae*	*K. pneumoniae*	1	NO	N/A
ARLG-3239	Yes	KPC3	NO	Negative	*K. pneumoniae*	*K. pneumoniae*	1	NO	N/A
ARLG-3240	Yes	KPC2	NO	Negative	*K. pneumoniae*	*K. pneumoniae*	1	NO	N/A
ARLG-3241	Yes	KPC2	YES	Positive	*K. pneumoniae*	*K. pneumoniae*	1	NO	N/A
ARLG-3242	Yes	KPC2	NO	Negative	*K. pneumoniae*	*K. pneumoniae*	1	NO	N/A
ARLG-3245	Yes	KPC2	YES	Positive	*K. pneumoniae*	*K. pneumoniae*	1	NO	N/A
ARLG-3247	Yes	KPC2	YES	Positive	*K. pneumoniae*	*K. pneumoniae*	1	NO	N/A
ARLG-3251	Yes	KPC2	YES	Positive	*K. pneumoniae*	*K. pneumoniae*	1	NO	N/A
ARLG-3254	Yes	KPC2	YES	Positive	*K. pneumoniae*	*K. pneumoniae*	1	NO	N/A
ARLG-3255	Yes	KPC2	NO	Negative	*K. pneumoniae*	*K. pneumoniae*	1	NO	N/A
ARLG-3256	Yes	KPC2	YES	Positive	*K. pneumoniae*	*K. pneumoniae*	1	NO	N/A
ARLG-3260	Yes	KPC2	YES	Positive	*K. pneumoniae*	*K. pneumoniae*	1	NO	N/A
ARLG-3261	Yes	KPC2	YES	Positive	*K. pneumoniae*	*K. pneumoniae*	1	YES	1
ARLG-3274	No	N/A	NO	Negative	*K. pneumoniae*	*K. pneumoniae*	1	NO	N/A
ARLG-3275	No	N/A	NO	Negative	*K. pneumoniae*	*K. pneumoniae*	1	NO	N/A
ARLG-3276	No	N/A	NO	Negative	*K. pneumoniae*	*K. pneumoniae*	1	NO	N/A
ARLG-3285	No	N/A	NO	Negative	*K. pneumoniae*	*K. pneumoniae*	1	NO	N/A
ARLG-3289	Yes	KPC2	NO	Negative	*K. pneumoniae*	*K. pneumoniae*	1	NO	N/A
ARLG-3294	Yes	KPC3	NO	Negative	*K. pneumoniae*	*K. pneumoniae*	1	NO	N/A
ARLG-3297	Yes	KPC3	NO	Negative	*K. pneumoniae*	*K. pneumoniae*	1	NO	N/A
ARLG-3303	Yes	KPC3	NO	Negative	*K. pneumoniae*	*K. pneumoniae*	1	NO	N/A
ARLG-3304	Yes	KPC2	YES	Positive	*K. pneumoniae*	*K. pneumoniae*	1	NO	N/A
ARLG-3310	Yes	KPC2	YES	Positive	*K. pneumoniae*	*K. pneumoniae*	1	NO	N/A

ARLG-3311	Yes	KPC2	YES	Positive	*K. pneumoniae*	*K. pneumoniae*	1	NO	N/A
ARLG-3312	Yes	KPC2	YES	Positive	*K. pneumoniae*	*K. pneumoniae*	1	NO	N/A
ARLG-3317	Yes	KPC2	NO	Negative	*K. pneumoniae*	*K. pneumoniae*	1	NO	N/A
ARLG-3319	Yes	KPC2	YES	Positive	*K. pneumoniae*	*K. pneumoniae*	1	NO	N/A
ARLG-3320	No	N/A	NO	Negative	*K. pneumoniae*	*K. pneumoniae*	1	NO	N/A
ARLG-3321	Yes	KPC2	NO	Negative	*K. pneumoniae*	*K. pneumoniae*	1	NO	N/A
ARLG-3322	Yes	KPC2	NO	Negative	*K. pneumoniae*	*K. pneumoniae*	1	NO	N/A
ARLG-3323	Yes	KPC3	NO	Negative	*K. pneumoniae*	*K. pneumoniae*	1	NO	N/A
ARLG-3328	Yes	KPC2	NO	Negative	*K. pneumoniae*	*K. pneumoniae*	1	NO	N/A
ARLG-3332	Yes	KPC2	YES	Positive	*K. pneumoniae*	*K. pneumoniae*	1	NO	N/A
ARLG-3340	No	N/A	NO	Negative	*K. pneumoniae*	*K. pneumoniae*	1	NO	N/A
ARLG-3342	Yes	KPC3	NO	Negative	*K. pneumoniae*	*K. pneumoniae*	1	NO	N/A
ARLG-3343	Yes	KPC3	NO	Negative	*K. pneumoniae*	*K. pneumoniae*	1	NO	N/A
ARLG-3344	Yes	KPC3	NO	Negative	*K. pneumoniae*	*K. pneumoniae*	1	NO	N/A
ARLG-3346	Yes	KPC3	NO	Negative	*K. pneumoniae*	*K. pneumoniae*	1	NO	N/A
ARLG-3348	Yes	KPC3	NO	Negative	*K. pneumoniae*	*K. pneumoniae*	1	NO	N/A
ARLG-3352	Yes	KPC3	NO	Negative	*K. pneumoniae*	*K. pneumoniae*	1	NO	N/A
ARLG-3353	Yes	KPC3	NO	Negative	*K. pneumoniae*	*K. pneumoniae*	1	NO	N/A
ARLG-3354	Yes	KPC3	NO	Negative	*K. pneumoniae*	*K. pneumoniae*	1	NO	N/A
ARLG-3355	No	N/A	NO	Negative	*K. pneumoniae*	*K. pneumoniae*	1	NO	N/A
ARLG-3357	No	N/A	NO	Negative	*K. pneumoniae*	*K. pneumoniae*	1	NO	N/A
ARLG-3358	Yes	KPC3	NO	Negative	*K. pneumoniae*	*K. pneumoniae*	1	NO	N/A
ARLG-3359	Yes	KPC3	NO	Negative	*K. pneumoniae*	*K. pneumoniae*	1	NO	N/A
ARLG-3361	Yes	KPC3	NO	Negative	*K. pneumoniae*	*K. pneumoniae*	1	NO	N/A
ARLG-3364	Yes	KPC2	YES	Positive	*K. pneumoniae*	*K. pneumoniae*	1	NO	N/A
ARLG-3365	Yes	KPC3	NO	Negative	*K. pneumoniae*	*K. pneumoniae*	1	NO	N/A
ARLG-3367	Yes	KPC3	NO	Negative	*K. pneumoniae*	*K. pneumoniae*	1	NO	N/A
ARLG-3368	Yes	KPC3	NO	Negative	*K. pneumoniae*	*K. pneumoniae*	1	NO	N/A
ARLG-3370	Yes	KPC2	YES	Positive	*K. pneumoniae*	*K. pneumoniae*	1	NO	N/A
ARLG-3371	Yes	KPC2	YES	Positive	*K. pneumoniae*	*K. pneumoniae*	1	NO	N/A

ARLG-3374	Yes	KPC3	NO	Negative	*K. pneumoniae*	*K. pneumoniae*	1	NO	N/A
ARLG-3375	Yes	KPC2	NO	Negative	*K. pneumoniae*	*K. pneumoniae*	1	NO	N/A
ARLG-3379	Yes	KPC2	YES	Positive	*K. pneumoniae*	*K. pneumoniae*	1	NO	N/A
ARLG-3381	No	N/A	NO	Negative	*K. pneumoniae*	*K. pneumoniae*	1	NO	N/A
ARLG-3383	Yes	KPC2	NO	Negative	*K. pneumoniae*	*K. pneumoniae*	1	NO	N/A
ARLG-3394	No	N/A	NO	Negative	*K. pneumoniae*	*K. pneumoniae*	1	NO	N/A
ARLG-3400	No	N/A	NO	Negative	*K. pneumoniae*	*K. pneumoniae*	1	NO	N/A
ARLG-3401	No	N/A	NO	Negative	*K. pneumoniae*	*K. pneumoniae*	1	NO	N/A
ARLG-3408	Yes	KPC2	YES	Positive	*K. pneumoniae*	*K. pneumoniae*	1	NO	N/A
ARLG-3412	Yes	KPC2	YES	Positive	*K. pneumoniae*	*K. pneumoniae*	1	NO	N/A
ARLG-3416	No	N/A	NO	Negative	*K. pneumoniae*	*K. pneumoniae*	1	NO	N/A
ARLG-3430	No	N/A	NO	Negative	*K. pneumoniae*	*K. pneumoniae*	1	NO	N/A
ARLG-3441	No	N/A	NO	Negative	*K. pneumoniae*	*K. pneumoniae*	1	NO	N/A
ARLG-3445	No	N/A	NO	Negative	*K. pneumoniae*	*K. pneumoniae*	1	NO	N/A
ARLG-3449	No	N/A	NO	Negative	*K. pneumoniae*	*K. pneumoniae*	1	NO	N/A
ARLG-3459	No	N/A	NO	Negative	*K. pneumoniae*	*K. pneumoniae*	1	NO	N/A
ARLG-3461	No	N/A	NO	Negative	*K. pneumoniae*	*K. pneumoniae*	1	NO	N/A
ARLG-3466	Yes	KPC3	NO	Negative	*K. pneumoniae*	*K. pneumoniae*	1	NO	N/A
ARLG-3474	No	N/A	NO	Negative	*K. pneumoniae*	*K. pneumoniae*	1	NO	N/A
ARLG-3475	No	N/A	NO	Negative	*K. pneumoniae*	*K. pneumoniae*	1	NO	N/A
ARLG-3479	Yes	KPC2	YES	Positive	*K. pneumoniae*	*K. pneumoniae*	1	NO	N/A
ARLG-3480	Yes	KPC2	YES	Positive	*K. pneumoniae*	*K. pneumoniae*	1	NO	N/A
ARLG-4565	Yes	KPC2	YES	Positive	*K. pneumoniae*	*K. pneumoniae*	1	NO	N/A
ARLG-4567	Yes	KPC2	YES	Positive	*K. pneumoniae*	*K. pneumoniae*	1	NO	N/A
ARLG-4568	Yes	KPC2	NO	Negative	*K. pneumoniae*	*K. pneumoniae*	1	NO	N/A
ARLG-4569	Yes	KPC2	YES	Positive	*K. pneumoniae*	*K. pneumoniae*	1	NO	N/A
ARLG-4570	Yes	KPC2	YES	Positive	*K. pneumoniae*	*K. pneumoniae*	1	NO	N/A
ARLG-4571	Yes	KPC2	YES	Positive	*K. pneumoniae*	*K. pneumoniae*	1	NO	N/A
ARLG-4574	No	N/A	NO	Negative	*K. pneumoniae*	*K. pneumoniae*	1	NO	N/A
ARLG-4575	Yes	KPC2	YES	Positive	*K. pneumoniae*	*K. pneumoniae*	1	NO	N/A

ARLG-4590	Yes	KPC2	YES	Positive	*K. pneumoniae*	*K. pneumoniae*	1	NO	N/A
ARLG-4591	Yes	KPC3	NO	Negative	*K. pneumoniae*	*K. pneumoniae*	1	NO	N/A
ARLG-4592	No	N/A	NO	Negative	*K. pneumoniae*	*K. pneumoniae*	1	NO	N/A
ARLG-4593	Yes	KPC3	NO	Negative	*K. pneumoniae*	*K. pneumoniae*	1	NO	N/A
ARLG-4595	Yes	KPC3	NO	Negative	*K. pneumoniae*	*K. pneumoniae*	1	NO	N/A
ARLG-4604	No	N/A	NO	Negative	*K. pneumoniae*	*K. pneumoniae*	1	NO	N/A
ARLG-4605	Yes	KPC2	YES	Positive	*K. pneumoniae*	*K. pneumoniae*	1	NO	N/A
ARLG-4606	No	N/A	NO	Negative	*K. pneumoniae*	*K. pneumoniae*	1	NO	N/A
ARLG-4608	No	N/A	NO	Negative	*K. pneumoniae*	*K. pneumoniae*	1	NO	N/A
ARLG-4609	No	N/A	NO	Negative	*K. pneumoniae*	*K. pneumoniae*	1	NO	N/A
ARLG-4612	Yes	KPC3	NO	Negative	*K. pneumoniae*	*K. pneumoniae*	1	NO	N/A
ARLG-4616	Yes	KPC3	NO	Negative	*K. pneumoniae*	*K. pneumoniae*	1	NO	N/A
ARLG-4617	Yes	KPC3	NO	Negative	*K. pneumoniae*	*K. pneumoniae*	1	NO	N/A
ARLG-4618	Yes	KPC3	NO	Negative	*K. pneumoniae*	*K. pneumoniae*	1	NO	N/A
ARLG-4619	Yes	KPC3	NO	Negative	*K. pneumoniae*	*K. pneumoniae*	1	NO	N/A
ARLG-4621	Yes	KPC3	NO	Negative	*K. pneumoniae*	*K. pneumoniae*	1	NO	N/A
ARLG-4622	Yes	KPC2	NO	Negative	*K. pneumoniae*	*K. pneumoniae*	1	NO	N/A
ARLG-4623	Yes	KPC2	YES	Positive	*K. pneumoniae*	*K. pneumoniae*	1	NO	N/A
ARLG-4624	Yes	KPC2	YES	Positive	*K. pneumoniae*	*K. pneumoniae*	1	NO	N/A
ARLG-4626	No	N/A	NO	Negative	*K. pneumoniae*	*K. pneumoniae*	1	NO	N/A
ARLG-4627	Yes	KPC3	NO	Negative	*K. pneumoniae*	*K. pneumoniae*	1	NO	N/A
ARLG-4632	No	N/A	NO	Negative	*K. pneumoniae*	*K. pneumoniae*	1	NO	N/A
ARLG-4633	Yes	KPC2	NO	Negative	*K. pneumoniae*	*K. pneumoniae*	1	NO	N/A
ARLG-4635	Yes	KPC3	NO	Negative	*K. pneumoniae*	*K. pneumoniae*	1	NO	N/A
ARLG-4637	Yes	KPC3	NO	Negative	*K. pneumoniae*	*K. pneumoniae*	1	NO	N/A
ARLG-4639	Yes	KPC3	NO	Negative	*K. pneumoniae*	*K. pneumoniae*	1	NO	N/A
ARLG-4640	Yes	KPC3	NO	Negative	*K. pneumoniae*	*K. pneumoniae*	1	NO	N/A
ARLG-4641	Yes	KPC3	NO	Negative	*K. pneumoniae*	*K. pneumoniae*	1	NO	N/A
ARLG-4642	Yes	KPC2	NO	Negative	*K. pneumoniae*	*K. pneumoniae*	1	NO	N/A
ARLG-4643	Yes	KPC3	NO	Negative	*K. pneumoniae*	*K. pneumoniae*	1	NO	N/A

ARLG-4645	Yes	KPC3	NO	Negative	*K. pneumoniae*	*K. pneumoniae*	1	NO	N/A
ARLG-4646	Yes	KPC2	NO	Negative	*K. pneumoniae*	*K. pneumoniae*	1	NO	N/A
ARLG-4647	Yes	KPC2	YES	Positive	*K. pneumoniae*	*K. pneumoniae*	1	NO	N/A
ARLG-4650	No	N/A	NO	Negative	*K. pneumoniae*	*K. pneumoniae*	1	NO	N/A
ARLG-4651	Yes	KPC3	NO	Negative	*K. pneumoniae*	*K. pneumoniae*	1	NO	N/A
ARLG-4653	Yes	KPC3	NO	Negative	*K. pneumoniae*	*K. pneumoniae*	1	NO	N/A
ARLG-4654	Yes	KPC3	NO	Negative	*K. pneumoniae*	*K. pneumoniae*	1	NO	N/A
ARLG-4655	Yes	KPC2	NO	Negative	*K. pneumoniae*	*K. pneumoniae*	1	NO	N/A
ARLG-4656	Yes	KPC3	NO	Negative	*K. pneumoniae*	*K. pneumoniae*	1	NO	N/A
ARLG-4657	Yes	KPC3	YES	Positive	*K. pneumoniae*	*K. pneumoniae*	1	NO	N/A
ARLG-4658	Yes	KPC3	NO	Negative	*K. pneumoniae*	*K. pneumoniae*	1	NO	N/A
ARLG-4661	No	N/A	NO	Negative	*K. pneumoniae*	*K. pneumoniae*	1	NO	N/A
ARLG-4662	Yes	KPC3	NO	Negative	*K. pneumoniae*	*K. pneumoniae*	1	NO	N/A
ARLG-4666	Yes	KPC2	NO	Negative	*K. pneumoniae*	*K. pneumoniae*	1	NO	N/A
ARLG-4667	Yes	KPC2	YES	Positive	*K. pneumoniae*	*K. pneumoniae*	1	NO	N/A
ARLG-4668	Yes	KPC2	YES	Positive	*K. pneumoniae*	*K. pneumoniae*	1	NO	N/A
ARLG-4669	No	N/A	NO	Negative	*K. pneumoniae*	*K. pneumoniae*	1	NO	N/A
ARLG-4670	No	N/A	NO	Negative	*K. pneumoniae*	*K. pneumoniae*	1	NO	N/A
ARLG-4671	Yes	KPC2	YES	Positive	*K. pneumoniae*	*K. pneumoniae*	1	NO	N/A
ARLG-4672	Yes	KPC3	NO	Negative	*K. pneumoniae*	*K. pneumoniae*	1	NO	N/A
ARLG-4673	Yes	KPC2	YES	Positive	*K. pneumoniae*	*K. pneumoniae*	1	NO	N/A
ARLG-4674	Yes	KPC3	NO	Negative	*K. pneumoniae*	*K. pneumoniae*	1	NO	N/A
ARLG-4675	Yes	KPC3	NO	Negative	*K. pneumoniae*	*K. pneumoniae*	1	NO	N/A
ARLG-4676	Yes	KPC3	NO	Negative	*K. pneumoniae*	*K. pneumoniae*	1	NO	N/A
ARLG-4682	Yes	KPC2	YES	Positive	*K. pneumoniae*	*K. pneumoniae*	1	NO	N/A
ARLG-4683	Yes	KPC2	YES	Positive	*K. pneumoniae*	*K. pneumoniae*	1	NO	N/A
ARLG-4684	Yes	KPC3	NO	Negative	*K. pneumoniae*	*K. pneumoniae*	1	NO	N/A
ARLG-4686	Yes	KPC3	NO	Negative	*K. pneumoniae*	*K. pneumoniae*	1	NO	N/A
ARLG-4691	Yes	KPC3	NO	Negative	*K. pneumoniae*	*K. pneumoniae*	1	NO	N/A
ARLG-4692	Yes	KPC3	NO	Negative	*K. pneumoniae*	*K. pneumoniae*	1	NO	N/A

ARLG-4693	Yes	KPC3	NO	Negative	*K. pneumoniae*	*K. pneumoniae*	1	NO	N/A
ARLG-4694	Yes	KPC3	NO	Negative	*K. pneumoniae*	*K. pneumoniae*	1	NO	N/A
ARLG-4696	Yes	KPC3	NO	Negative	*K. pneumoniae*	*K. pneumoniae*	1	NO	N/A
ARLG-4697	Yes	KPC2	YES	Positive	*K. pneumoniae*	*K. pneumoniae*	1	NO	N/A
ARLG-4698	No	N/A	NO	Negative	*K. pneumoniae*	*K. pneumoniae*	1	NO	N/A
ARLG-4703	Yes	KPC2	NO	Negative	*K. pneumoniae*	*K. pneumoniae*	1	NO	N/A
ARLG-4704	Yes	KPC3	NO	Negative	*K. pneumoniae*	*K. pneumoniae*	1	NO	N/A
ARLG-4706	Yes	KPC2	NO	Negative	*K. pneumoniae*	*K. pneumoniae*	1	NO	N/A
ARLG-4707	Yes	KPC2	YES	Positive	*K. pneumoniae*	*K. pneumoniae*	1	NO	N/A
ARLG-4709	Yes	KPC3	NO	Negative	*K. pneumoniae*	*K. pneumoniae*	1	NO	N/A
ARLG-4710	Yes	KPC2	NO	Negative	*K. pneumoniae*	*K. pneumoniae*	1	NO	N/A
ARLG-4711	No	N/A	NO	Negative	*K. pneumoniae*	*K. pneumoniae*	1	NO	N/A
ARLG-4712	No	N/A	NO	Negative	*K. pneumoniae*	*K. pneumoniae*	1	NO	N/A
ARLG-4716	Yes	KPC3	NO	Negative	*K. pneumoniae*	*K. pneumoniae*	1	NO	N/A
ARLG-4720	No	N/A	NO	Negative	*K. pneumoniae*	*K. pneumoniae*	1	NO	N/A
ARLG-4723	Yes	KPC2	NO	Negative	*K. pneumoniae*	*K. pneumoniae*	1	NO	N/A
ARLG-4728	No	N/A	NO	Negative	*K. pneumoniae*	*K. pneumoniae*	1	NO	N/A
ARLG-4729	No	N/A	NO	Negative	*K. pneumoniae*	*K. pneumoniae*	1	NO	N/A
ARLG-4730	No	N/A	NO	Negative	*K. pneumoniae*	*K. pneumoniae*	1	NO	N/A
ARLG-4743	No	N/A	NO	Negative	*K. pneumoniae*	*K. pneumoniae*	1	NO	N/A
ARLG-4744	No	N/A	NO	Negative	*K. pneumoniae*	*K. pneumoniae*	1	NO	N/A
ARLG-4747	No	N/A	NO	Negative	*K. pneumoniae*	*K. pneumoniae*	1	NO	N/A
ARLG-4749	Yes	KPC2	NO	Negative	*K. pneumoniae*	*K. pneumoniae*	1	NO	N/A
ARLG-4751	Yes	KPC2	NO	Negative	*K. pneumoniae*	*K. pneumoniae*	1	NO	N/A
ARLG-4752	Yes	KPC3	NO	Negative	*K. pneumoniae*	*K. pneumoniae*	1	NO	N/A
ARLG-4753	Yes	KPC2	NO	Negative	*K. pneumoniae*	*K. pneumoniae*	1	NO	N/A
ARLG-4756	Yes	KPC2	NO	Negative	*K. pneumoniae*	*K. pneumoniae*	1	NO	N/A
ARLG-4758	Yes	KPC3	NO	Negative	*K. pneumoniae*	*K. pneumoniae*	1	NO	N/A
ARLG-4759	No	N/A	NO	Negative	*K. pneumoniae*	*K. pneumoniae*	1	NO	N/A
ARLG-4760	Yes	KPC3	NO	Negative	*K. pneumoniae*	*K. pneumoniae*	1	NO	N/A

ARLG-4764	No	N/A	NO	Negative	*K. pneumoniae*	*K. pneumoniae*	1	NO	N/A
ARLG-4766	Yes	KPC3	NO	Negative	*K. pneumoniae*	*K. pneumoniae*	1	NO	N/A
ARLG-4767	No	N/A	NO	Negative	*K. pneumoniae*	*K. pneumoniae*	1	NO	N/A
ARLG-4768	No	N/A	NO	Negative	*K. pneumoniae*	*K. pneumoniae*	1	NO	N/A
ARLG-4771	No	N/A	NO	Negative	*K. pneumoniae*	*K. pneumoniae*	1	NO	N/A
ARLG-4777	No	N/A	NO	Negative	*K. pneumoniae*	*K. pneumoniae*	1	NO	N/A
ARLG-4783	No	N/A	NO	Negative	*K. pneumoniae*	*K. pneumoniae*	1	NO	N/A
ARLG-4793	No	N/A	NO	Negative	*K. pneumoniae*	*K. pneumoniae*	1	NO	N/A
ARLG-4794	Yes	KPC3	NO	Negative	*K. pneumoniae*	*K. pneumoniae*	1	NO	N/A
ARLG-4796	No	N/A	NO	Negative	*K. pneumoniae*	*K. pneumoniae*	1	NO	N/A
ARLG-4798	Yes	KPC2	NO	Negative	*K. pneumoniae*	*K. pneumoniae*	1	NO	N/A
ARLG-4802	No	N/A	NO	Negative	*K. pneumoniae*	*K. pneumoniae*	1	NO	N/A
ARLG-4803	No	N/A	NO	Negative	*K. pneumoniae*	*K. pneumoniae*	1	NO	N/A
ARLG-4806	Yes	KPC3	NO	Negative	*K. pneumoniae*	*K. pneumoniae*	1	NO	N/A
ARLG-4807	Yes	KPC2	YES	Positive	*K. pneumoniae*	*K. pneumoniae*	1	YES	2
ARLG-4808	No	N/A	NO	Negative	*K. pneumoniae*	*K. pneumoniae*	1	NO	N/A
ARLG-4809	Yes	KPC3	NO	Negative	*K. pneumoniae*	*K. pneumoniae*	1	NO	N/A
ARLG-4810	Yes	KPC2	YES	Positive	*K. pneumoniae*	*K. pneumoniae*	1	NO	N/A
ARLG-4812	Yes	KPC2	YES	Positive	*K. pneumoniae*	*K. pneumoniae*	1	NO	N/A
ARLG-4814	Yes	KPC2	NO	Negative	*K. pneumoniae*	*K. pneumoniae*	1	NO	N/A
ARLG-4815	Yes	KPC2	YES	Positive	*K. pneumoniae*	*K. pneumoniae*	1	YES	1
ARLG-4817	Yes	KPC3	NO	Negative	*K. pneumoniae*	*K. pneumoniae*	1	NO	N/A
ARLG-4818	Yes	KPC2	YES	Positive	*K. pneumoniae*	*K. pneumoniae*	1	NO	N/A
ARLG-4819	Yes	KPC2	YES	Positive	*K. pneumoniae*	*K. pneumoniae*	1	NO	N/A
ARLG-4830	Yes	KPC3	NO	Negative	*K. pneumoniae*	*K. pneumoniae*	1	NO	N/A
ARLG-4832	Yes	KPC2	NO	Negative	*K. pneumoniae*	*K. pneumoniae*	1	NO	N/A
ARLG-4833	Yes	KPC2	NO	Negative	*K. pneumoniae*	*K. pneumoniae*	1	NO	N/A
ARLG-4834	Yes	KPC2	YES	Positive	*K. pneumoniae*	*K. pneumoniae*	1	NO	N/A
ARLG-4842	Yes	KPC2	YES	Positive	*K. pneumoniae*	*K. pneumoniae*	1	NO	N/A
ARLG-4843	Yes	KPC2	YES	Positive	*K. pneumoniae*	*K. pneumoniae*	1	NO	N/A

ARLG-4844	No	N/A	NO	Negative	*K. pneumoniae*	*K. pneumoniae*	1	NO	N/A
ARLG-4847	Yes	KPC2	YES	Positive	*K. pneumoniae*	*K. pneumoniae*	1	NO	N/A
ARLG-4849	Yes	KPC2	YES	Positive	*K. pneumoniae*	*K. pneumoniae*	1	NO	N/A
ARLG-4850	No	N/A	NO	Negative	*K. pneumoniae*	*K. pneumoniae*	1	NO	N/A
ARLG-4851	No	N/A	NO	Negative	*K. pneumoniae*	*K. pneumoniae*	1	NO	N/A
ARLG-4855	Yes	KPC2	YES	Positive	*K. pneumoniae*	*K. pneumoniae*	1	NO	N/A
ARLG-4856	Yes	KPC2	YES	Positive	*K. pneumoniae*	*K. pneumoniae*	1	NO	N/A
ARLG-4857	Yes	KPC2	YES	Positive	*K. pneumoniae*	*K. pneumoniae*	1	NO	N/A
ARLG-4859	Yes	KPC2	YES	Positive	*K. pneumoniae*	*K. pneumoniae*	1	NO	N/A
ARLG-4860	Yes	KPC2	YES	Positive	*K. pneumoniae*	*K. pneumoniae*	1	NO	N/A
ARLG-4861	Yes	KPC3	NO	Negative	*K. pneumoniae*	*K. pneumoniae*	1	NO	N/A
ARLG-4864	Yes	KPC2	YES	Positive	*K. pneumoniae*	*K. pneumoniae*	1	NO	N/A
ARLG-4865	No	N/A	NO	Negative	*K. pneumoniae*	*K. pneumoniae*	1	NO	N/A
ARLG-4866	Yes	KPC2	YES	Positive	*K. pneumoniae*	*K. pneumoniae*	1	NO	N/A
ARLG-4870	Yes	KPC2	YES	Positive	*K. pneumoniae*	*K. pneumoniae*	1	YES	1
ARLG-4871	Yes	KPC2	YES	Negative	*K. pneumoniae*	*K. pneumoniae*	1	NO	N/A
ARLG-4872	Yes	KPC2	YES	Positive	*K. pneumoniae*	*K. pneumoniae*	1	YES	1
ARLG-4875	Yes	KPC2	YES	Positive	*K. pneumoniae*	*K. pneumoniae*	1	NO	N/A
ARLG-4877	Yes	KPC2	YES	Positive	*K. pneumoniae*	*K. pneumoniae*	1	NO	N/A
ARLG-4878	Yes	KPC2	YES	Positive	*K. pneumoniae*	*K. pneumoniae*	1	NO	N/A
ARLG-4879	Yes	KPC2	NO	Negative	*K. pneumoniae*	*K. pneumoniae*	1	NO	N/A
ARLG-4880	Yes	KPC2	YES	Positive	*K. pneumoniae*	*K. pneumoniae*	1	NO	N/A
ARLG-4882	Yes	KPC2	YES	Positive	*K. pneumoniae*	*K. pneumoniae*	1	NO	N/A
ARLG-4883	Yes	KPC3	NO	Negative	*K. pneumoniae*	*K. pneumoniae*	1	NO	N/A
ARLG-4884	Yes	KPC3	NO	Negative	*K. pneumoniae*	*K. pneumoniae*	1	NO	N/A
ARLG-4885	Yes	KPC2	YES	Positive	*K. pneumoniae*	*K. pneumoniae*	1	NO	N/A
ARLG-4886	Yes	KPC3	NO	Negative	*K. pneumoniae*	*K. pneumoniae*	1	NO	N/A
ARLG-4887	Yes	KPC2	YES	Positive	*K. pneumoniae*	*K. pneumoniae*	1	NO	N/A
ARLG-4888	Yes	KPC2	YES	Positive	*K. pneumoniae*	*K. pneumoniae*	1	NO	N/A
ARLG-4889	Yes	KPC2	YES	Positive	*K. pneumoniae*	*K. pneumoniae*	1	NO	N/A

ARLG-4890	Yes	KPC2	YES	Positive	*K. pneumoniae*	*K. pneumoniae*	1	NO	N/A
ARLG-4891	Yes	KPC3	NO	Negative	*K. pneumoniae*	*K. pneumoniae*	1	NO	N/A
ARLG-4892	Yes	KPC3	NO	Negative	*K. pneumoniae*	*K. pneumoniae*	1	NO	N/A
ARLG-4893	Yes	KPC2	YES	Positive	*K. pneumoniae*	*K. pneumoniae*	1	NO	N/A
ARLG-4896	Yes	KPC2	YES	Positive	*K. pneumoniae*	*K. pneumoniae*	1	NO	N/A
ARLG-4898	Yes	KPC3	NO	Negative	*K. pneumoniae*	*K. pneumoniae*	1	NO	N/A
ARLG-4899	Yes	KPC3	NO	Negative	*K. pneumoniae*	*K. pneumoniae*	1	NO	N/A
ARLG-4903	Yes	KPC2	YES	Positive	*K. pneumoniae*	*K. pneumoniae*	1	NO	N/A
ARLG-4906	No	N/A	NO	Negative	*K. pneumoniae*	*K. pneumoniae*	1	NO	N/A
ARLG-4907	Yes	KPC3	NO	Negative	*K. pneumoniae*	*K. pneumoniae*	1	NO	N/A
ARLG-4908	No	N/A	NO	Negative	*K. pneumoniae*	*K. pneumoniae*	1	NO	N/A
ARLG-4912	Yes	KPC3	NO	Negative	*K. pneumoniae*	*K. pneumoniae*	1	NO	N/A
ARLG-4915	Yes	KPC2	YES	Positive	*K. pneumoniae*	*K. pneumoniae*	1	NO	N/A
ARLG-4916	Yes	KPC2	YES	Positive	*K. pneumoniae*	*K. pneumoniae*	1	NO	N/A
ARLG-4917	Yes	KPC3	NO	Negative	*K. pneumoniae*	*K. pneumoniae*	1	NO	N/A
ARLG-4918	Yes	KPC3	NO	Negative	*K. pneumoniae*	*K. pneumoniae*	1	NO	N/A
ARLG-4920	Yes	KPC3	NO	Negative	*K. pneumoniae*	*K. pneumoniae*	1	NO	N/A
ARLG-4921	Yes	KPC2	YES	Positive	*K. pneumoniae*	*K. pneumoniae*	1	NO	N/A
ARLG-4922	Yes	KPC2	YES	Positive	*K. pneumoniae*	*K. pneumoniae*	1	NO	N/A
ARLG-4926	No	N/A	NO	Negative	*K. pneumoniae*	*K. pneumoniae*	1	NO	N/A
ARLG-4928	Yes	KPC3	NO	Negative	*K. pneumoniae*	*K. pneumoniae*	1	NO	N/A
ARLG-4929	Yes	KPC2	YES	Positive	*K. pneumoniae*	*K. pneumoniae*	1	NO	N/A
ARLG-4930	Yes	KPC3	NO	Negative	*K. pneumoniae*	*K. pneumoniae*	1	NO	N/A
ARLG-4932	No	N/A	NO	Negative	*K. pneumoniae*	*K. pneumoniae*	1	NO	N/A

*1 USA, 2 Central/Northern North America (Colombia/Nicaragua), 3 Southern South America (Argentina/Chile), 4 Asia-Pacific (Lebanon/Singapore).

**1 Resolved KPC detection, 2 Persistent discrepancy, 3 Persistent identification score <2.0, 4 Resolved identification score ≥2.0
