# Supplementary figures and images for: Poor Sensitivity of the MALDI Biotyper® MBT Subtyping Module for Detection of Klebsiella pneumoniae Carbapenemase (KPC) in Klebsiella Species
Source: Antibiotics (Basel). 2023 Sep 20;12(9):1465. doi: 10.3390/antibiotics12091465 (PMC10525285; doi:10.3390/antibiotics12091465)

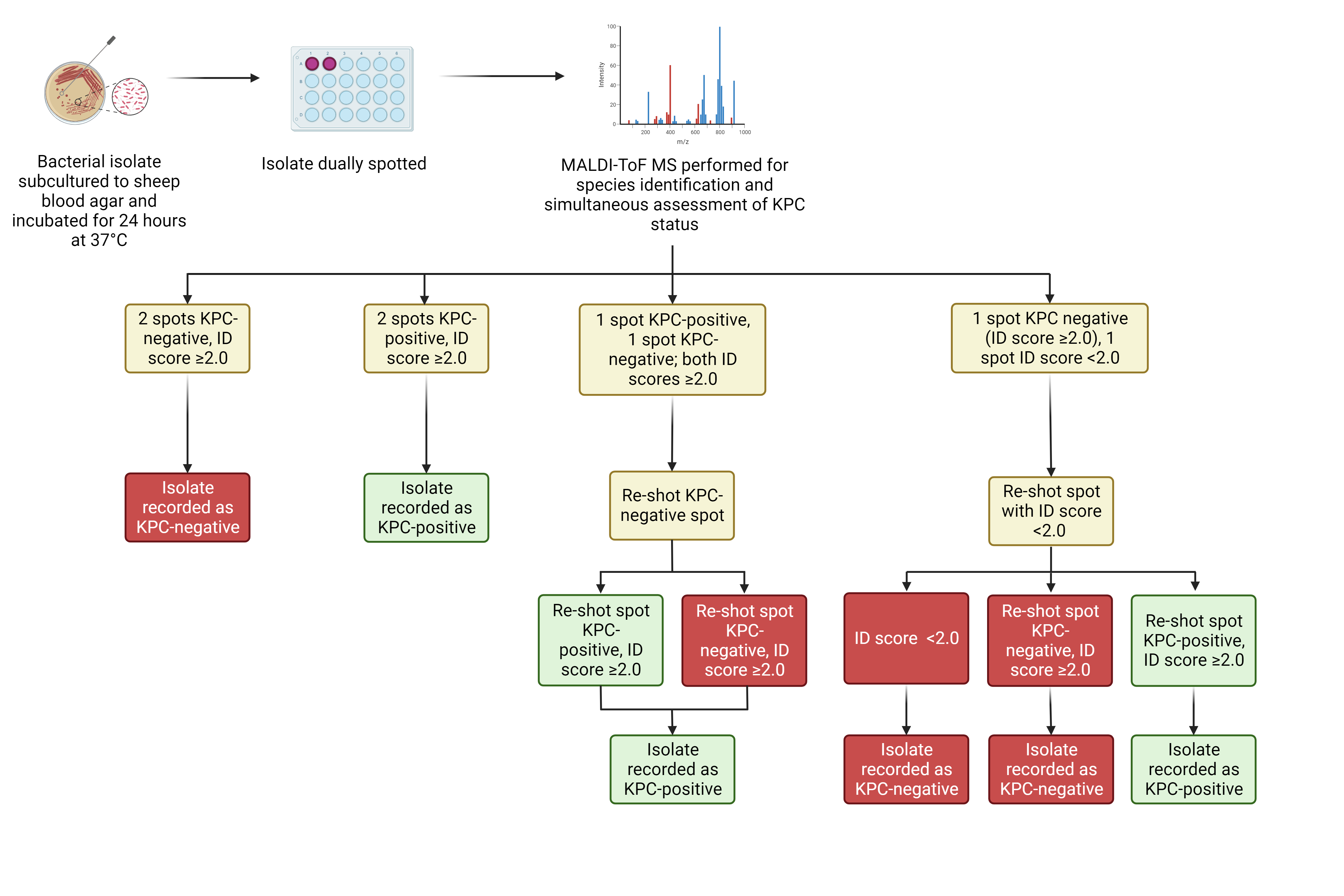

Supplement: Supplementary file 1 [file antibiotics-12-01465-s001.zip › Figure S1- Poor sensitivity of the MALDI Biotyper MBT Subtyping Module.png]
